# Dynamic Changes in the MicroRNA Expression Profile Reveal Multiple Regulatory Mechanisms in the Spinal Nerve Ligation Model of Neuropathic Pain

**DOI:** 10.1371/journal.pone.0017670

**Published:** 2011-03-14

**Authors:** David von Schack, Michael J. Agostino, B. Stuart Murray, Yizheng Li, Padmalatha S. Reddy, Jin Chen, Sung E. Choe, Brian W. Strassle, Christine Li, Brian Bates, Lynn Zhang, Huijuan Hu, Smita Kotnis, Brendan Bingham, Wei Liu, Garth T. Whiteside, Tarek A. Samad, Jeffrey D. Kennedy, Seena K. Ajit

**Affiliations:** 1 Biological Technologies, Pfizer Global Research and Development, Cambridge, Massachusetts, United States of America; 2 Neuroscience Discovery, Pfizer Global Research and Development, Princeton, New Jersey, United States of America; 3 Department of Pharmacology & Physiology, Drexel University College of Medicine, Philadelphia, Pennsylvania, United States of America; University of California, Los Angeles, and Cedars-Sinai Medical Center, United States of America

## Abstract

Neuropathic pain resulting from nerve lesions or dysfunction represents one of the most challenging neurological diseases to treat. A better understanding of the molecular mechanisms responsible for causing these maladaptive responses can help develop novel therapeutic strategies and biomarkers for neuropathic pain. We performed a miRNA expression profiling study of dorsal root ganglion (DRG) tissue from rats four weeks post spinal nerve ligation (SNL), a model of neuropathic pain. TaqMan low density arrays identified 63 miRNAs whose level of expression was significantly altered following SNL surgery. Of these, 59 were downregulated and the ipsilateral L4 DRG, not the injured L5 DRG, showed the most significant downregulation suggesting that miRNA changes in the uninjured afferents may underlie the development and maintenance of neuropathic pain. TargetScan was used to predict mRNA targets for these miRNAs and it was found that the transcripts with multiple predicted target sites belong to neurologically important pathways. By employing different bioinformatic approaches we identified neurite remodeling as a significantly regulated biological pathway, and some of these predictions were confirmed by siRNA knockdown for genes that regulate neurite growth in differentiated Neuro2A cells. *In vitro* validation for predicted target sites in the 3′-UTR of voltage-gated sodium channel *Scn11a*, alpha 2/delta1 subunit of voltage-dependent Ca-channel, and purinergic receptor *P2rx* ligand-gated ion channel 4 using luciferase reporter assays showed that identified miRNAs modulated gene expression significantly. Our results suggest the potential for miRNAs to play a direct role in neuropathic pain.

## Introduction

Neuropathic pain arises from nerve damage or dysfunction, adversely impacting quality of life and imposing a large healthcare burden [1], [2], [3]. A deeper understanding of the molecular mechanisms underlying neuropathic pain could provide a first step toward the development of better treatment options for patients. A frequently used rat model to study the molecular mechanisms of neuropathic pain is spinal nerve ligation (SNL) wherein one or more spinal nerves innervating the hind limb are ligated [4], typically unilaterally. The injury, which results in hyperalgesia, an enhanced response to mechanical stimuli, has a well-characterized time course. Since they represent a primary site for pain processing, dorsal root ganglion (DRG) neurons have been the focus of much research to identify molecular targets of pain neurotransmission. Previous studies using animal pain models have measured mRNA levels by examining a targeted set of transcripts or through the use of global approaches such as microarray technology to study mRNA expression changes [5], [6]. In a proteomic study, 67 proteins have been shown to be regulated in the SNL model [7].

MicroRNA (miRNA), a class of ∼22 nucleotide, non-protein encoding endogenous RNA molecules, has attracted considerable attention recently for its role in the molecular changes underlying various disease models [8]. miRNAs participate in the regulation of gene expression by binding to the 3′ untranslated region (3′-UTR) of target mRNAs, which can result in reduced expression of the proteins encoded by such target RNAs. Reduction of protein expression can come about by either of two mechanisms, the cleavage and degradation of the mRNA target or repression of its translation. Under the former mechanism but not the latter, an inverse correlation between miRNA and target mRNA expression is expected. Each miRNA species regulates multiple genes, and most mRNAs contain multiple miRNA binding sites within their 3′-UTR, suggestive of a complex regulatory network [9]. Since aberrant miRNA expression is a common feature in a variety of human diseases, an understanding of the gene regulation events in neuropathic pain mediated by miRNAs could provide an avenue for the identification of biomarkers or discovery of novel therapeutic targets [8]. We performed a miRNA expression profiling study of DRG 4 weeks following SNL surgery. A combination of bioinformatics and experimental approaches, including comparison to mRNA microarray profiling performed on the same biological samples, were applied to identify biological functions affected by the observed changes in miRNA expression.

## Results

**Figure 1:**
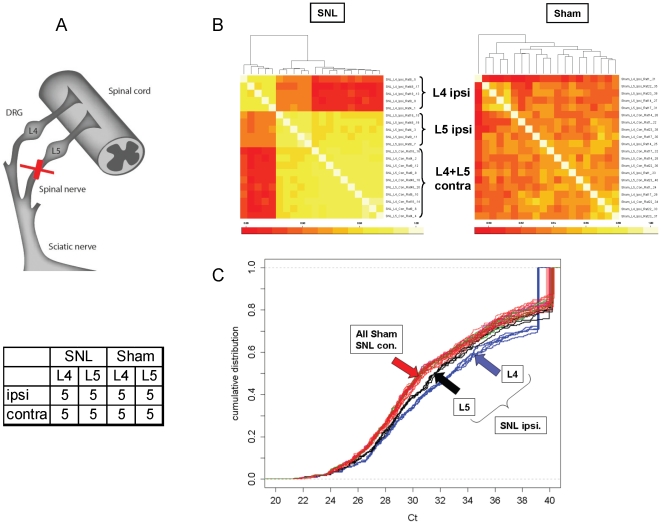
Profiling of miRNAs in DRG. **a. Schematic representation of spinal L5 nerve ligation adapted from Decosterd and Woolf (Pain, 2000).** Individual L4 and L5 DRG tissue samples were collected from the ipsi- and contralateral sides of 5 sham (no ligature) surgery and 5 SNL surgery rats four weeks after SNL for analysis. **b. Pair-wise correlation of raw** ***C*** **_T_ values among DRG samples.** Heat map of sample-to-sample (Pearson) correlation of raw *C*_T_ values from 248 detectable miRNAs in 40 DRG samples from the 8 experimental groups. Column and row labels are symmetric. **c. Distribution of normalized** ***C*** **_T_ values (Δ** ***C*** **_T_) in 40 samples.** Each line represents *C*_T_ distributions from one particular sample. The color represents the experimental group. L4 and L5 ipsilateral DRGs in the SNL cohort (blue and black, respectively) are clearly separated from each other and from the rest of the groups which appear to form an aggregate ‘group’ that contains all sham samples and all SNL contralateral samples. The majority of data points (*C*_T_ ranging from 29 to 39) in the blue curves are “shifted” to the right of the rest of the DRG sample distributions, indicating a general down-regulation (hence higher *C*_T_) of miRNA expression in L4 ipsilateral DRGs of the SNL cohort.

The SNL model has been used widely to investigate neuropathic pain mechanisms and as an assay for the development of new analgesic drugs. Ligation of spinal nerve results in long-lasting behavioral signs of mechanical hyperalgesia and tactile allodynia. Here we used the ligature of L5 (Figure 1a) and tested for tactile allodynia to confirm the success of the surgery in eliciting a pain response (data not shown).

### MicroRNA data quality assessment, normalization and statistical analysis

L4 and L5 dorsal root ganglia were collected from the ipsi- and contralateral sides of SNL and sham-operated rats. A Taqman Low Density Array (TLDA) miRNA expression profiling study was performed on RNA extracted from these DRG samples. Heatmap representation of sample to sample correlations shows a distinct separation of all L4 ipsilateral samples from the L4 and L5 contralateral samples, which are not separated from each other. The SNL L5 ipsilateral samples lie in between. Absence of separation of the samples in the sham group indicates no systematic effect of sham surgery on miRNA levels in this group (Figure 1b).

Normalization of the miRNA dataset was performed using the 11 most rank-preserving miRNAs across all 40 samples (Figure S1). The plot of normalized *C*_T_ distributions (Figure 1c) again shows a pattern that is consistent with the heatmap display: a very distinct grouping of L4, and to a lesser degree L5 ipsilateral DRGs from the SNL cohort from each other and from the rest of the samples. These patterns indicate that this pain model resulted in SNL-specific changes in miRNA expression levels, suggestive of an SNL- injury driven alteration in the underlying molecular biology of the DRGs.

A statistical analysis of the miRNAs that were differentially expressed in L4 DRG samples was conducted by applying a filter of *p*-value<0.01 and at least a 2-fold change in either direction. This resulted in a list of 63 miRNAs (henceforth referred to as the “63-set”) among the 365 miRNAs that were interrogated. A set of 10 microRNAs (the “10-set”) appear to turn off their expression to nearly undetectable levels in L4 ipsilateral DRGs, resulting in very large expression fold changes (up to ∼900 fold). Table 1 lists the 63-set miRNAs along with their *p*-values and signed fold changes (signFC) for L4 and L5 segments, which shows mostly downregulation of miRNA expression in ipsilateral L4 DRGs. Although the SNL effects on miRNA expression are more profound in the L4 DRG samples, 45 members of the 63-set meet the criteria for being differentially expressed in L5 as well.

**Table 1:** Sixty three miRNAs significantly altered following SNL surgery.

miRNA	SNL L4 FC (ips/con)	SNL L4 *p*-value (t-test)	SNL L5 FC (ips/con)	SNL L5 *p*-value (t-test)	# Predicted Targets	60th Percentile Context Score
**hsa-miR-221**	**−921.8**	1.3E-07	−1.3	9.9E-03	236	174
**hsa-miR-34a**	**−604.4**	1.0E-03	**−14.9**	9.6E-04	409	269
**hsa-let-7e**	**−337.7**	1.4E-06	**−22.6**	7.0E-03	758	403
**hsa-miR-132**	**−234.1**	6.0E-05	−2.4	3.7E-02	232	151
**hsa-miR-378**	**−185.7**	1.1E-03	−2.4	5.7E-02	84	57
**hsa-miR-34c**	**−115.9**	2.7E-04	−2.6	4.6E-02	409	269
**hsa-miR-409-5p**	**−104.4**	7.0E-04	−2.5	5.3E-02	70	43
**hsa-miR-135a**	**−65.8**	2.1E-03	−15.1	2.1E-02	426	253
**hsa-miR-18a**	**−35.6**	7.7E-03	−3.0	1.3E-01	139	110
**hsa-miR-17-3p**	**−16.2**	2.4E-03	−3.6	1.6E-01	165	119
hsa-let-7a	**−459.7**	2.8E-04	**−9.7**	7.5E-06	758	403
hsa-miR-21	**−382.2**	2.6E-03	**−2.2**	1.5E-04	167	112
hsa-miR-10b	**−309.5**	9.6E-04	**−8.5**	4.8E-05	54	39
hsa-let-7d	**−259.1**	3.5E-03	**−5.8**	4.1E-05	758	403
hsa-miR-93	**−208.8**	4.4E-03	**−2.4**	4.5E-05	846	550
hsa-miR-20a	**−177.7**	3.4E-03	−1.2	5.2E-02	846	550
hsa-miR-497	**−132.0**	7.1E-03	**−5.3**	1.2E-03	936	528
hsa-let-7b	**−89.3**	1.6E-06	**−5.9**	3.0E-06	758	403
hsa-miR-10a	**−84.8**	1.9E-04	**−4.8**	1.6E-04	147	107
hsa-let-7c	**−80.7**	3.8E-07	**−6.5**	1.0E-05	758	403
hsa-miR-142-3p	**−69.0**	5.7E-06	−1.9	7.4E-04	217	152
hsa-miR-27b	**−58.1**	3.6E-06	**−6.9**	2.4E-07	792	524
hsa-let-7g	**−50.1**	5.5E-05	**−4.9**	1.6E-05	**	**
hsa-miR-301	**−45.3**	1.6E-05	−1.8	2.9E-04	576	353
hsa-miR-324-5p	**−39.8**	6.3E-03	**−3.7**	4.3E-05	55	38
hsa-miR-133a	**−24.6**	3.8E-04	**−12.0**	1.8E-05	429	228
hsa-miR-20b	**−21.3**	8.5E-04	−1.1	4.7E-01	846	550
hsa-miR-125b	**−19.4**	1.2E-07	**−3.2**	2.9E-08	520	337
hsa-miR-27a	**−16.0**	3.3E-06	**−2.3**	3.7E-05	792	524
hsa-miR-148b	**−15.6**	1.9E-05	**−2.1**	1.4E-03	437	319
hsa-miR-369-5p	**−15.5**	1.8E-03	**−2.4**	3.1E-03	5	3
hsa-miR-92	**−12.1**	2.9E-05	−1.3	1.5E-02	597	348
hsa-miR-181c	**−11.6**	6.9E-03	**−2.3**	1.7E-03	767	467
hsa-miR-100	**−9.6**	8.3E-07	**−2.0**	2.4E-05	37	28
hsa-miR-148a	**−8.5**	5.9E-06	**−3.0**	9.5E-06	437	319
hsa-miR-383	**−7.0**	1.1E-03	**−3.7**	1.7E-03	79	48
hsa-miR-9	**−7.0**	4.2E-07	**−3.8**	2.7E-05	892	501
hsa-miR-127	**−6.3**	2.3E-06	−1.7	1.5E-05	6	5
hsa-miR-26b	**−6.0**	5.3E-06	**−2.0**	5.4E-05	588	377
hsa-miR-30a-5p	**−5.9**	4.1E-06	−1.3	8.2E-03	1026	593
hsa-miR-142-5p	**−5.4**	1.4E-03	1.0	8.7E-01	427	238
hsa-miR-30d	**−5.3**	1.6E-06	6.5	4.1E-01	1026	593
hsa-miR-190	**−4.3**	6.6E-03	**−4.2**	1.6E-05	84	57
hsa-miR-19a	**−4.1**	2.9E-06	1.7	1.2E-03	791	516
hsa-miR-23b	**−4.0**	6.2E-05	**−3.8**	1.8E-04	713	487
hsa-miR-339	**−3.9**	3.0E-04	−3.5	2.3E-02	83	49
hsa-miR-137	**−3.7**	7.3E-06	**−5.3**	7.0E-07	758	379
hsa-miR-181b	**−3.7**	2.9E-04	**−2.0**	6.3E-06	767	467
hsa-miR-19b	**−3.6**	1.0E-04	1.7	8.3E-05	791	516
hsa-miR-126	**−3.3**	3.2E-07	−1.7	3.6E-05	16	10
hsa-miR-218	**−3.2**	1.2E-05	−1.0	3.8E-01	635	393
hsa-miR-181d	**−3.0**	8.8E-04	−1.3	1.2E-02	767	467
hsa-miR-335	**−2.7**	6.2E-04	**−2.7**	9.7E-05	121	88
hsa-miR-103	**−2.7**	7.9E-05	**−2.0**	2.1E-04	360	243
hsa-miR-26a	**−2.7**	1.6E-05	−1.5	2.2E-03	588	377
hsa-miR-299-5p	**−2.6**	2.9E-03	−1.3	4.2E-02	129	68
hsa-miR-572	**−2.6**	6.2E-03	**−3.4**	6.5E-03	**	**
hsa-miR-659	**−2.5**	1.2E-03	−2.2	7.1E-02	**	**
hsa-miR-338	**−2.3**	2.9E-04	**−3.0**	5.5E-04	154	100
hsa-miR-486	**2.1**	1.0E-03	−1.6	2.6E-03	**	**
hsa-miR-30a-3p	**2.5**	6.1E-04	1.4	1.0E-03	**	**
hsa-miR-206	**4.3**	3.9E-05	1.3	1.3E-02	511	338
hsa-miR-133b	**4.6**	1.1E-06	**10.2**	7.3E-05	429	228

The set of 63 miRNAs found to be most differentially expressed in L4 ipsilateral DRGs of the SNL cohort compared to their contralateral counterpart. The set was derived based on the filters of t-test *p*-value<0.01 (FDR<2%) and fold change (at least two fold in either direction). The majority of these miRNAs were down-regulated in the ipsilateral relative to the contralateral DRGs, including the set of 10 miRNAs (bold type, top section of the table) whose expression level was almost undetectable in L4 ipsilateral DRGs (see text). The table was sorted by signed fold change (FC) for SNL L4. Bold type numbers indicate at least 2 fold up- or down-regulation in ipsilateral relative to contralateral DRGs. Number of mRNA transcripts containing predicted target sites for these 63-set miRNAs based on TargetScan predictions is shown. For increasing the stringency of the predicted target sites of the 63 miRNAs, we applied a context score filter above the 60^th^ percentile.

**No prediction from TargetScan database.

### *In vitro* validation using a reporter assay

**Figure 2:**
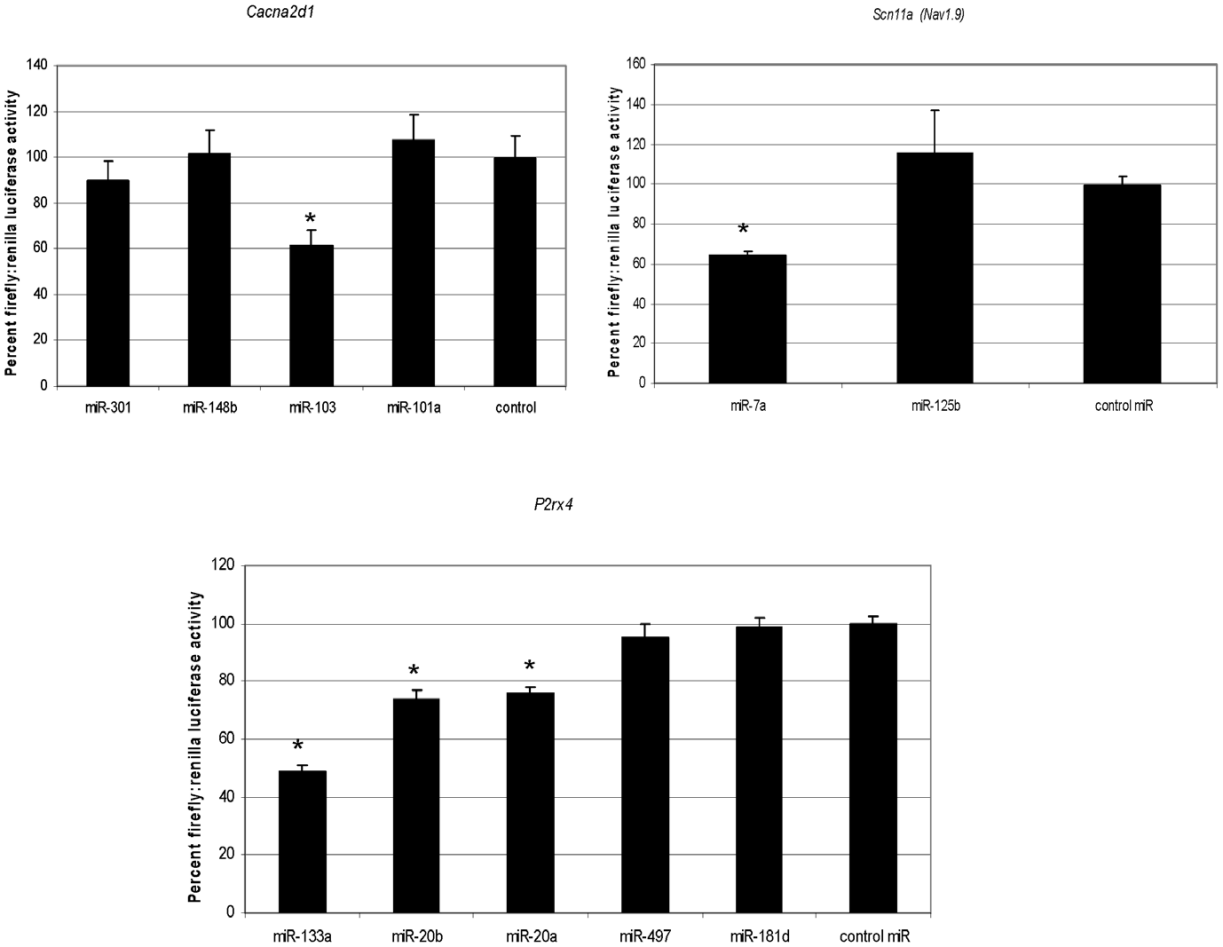
Luciferase reporter gene assay to assess the effect of miRNA on gene function. The effect of miRNA on gene regulation was monitored by co-transfecting 100 nM miRNA and the luciferase reporter gene. The 3′-UTR of *Scn11a* (*Nav1.9*), *Cacna2d1*and *P2rx4* were cloned downstream of the luciferase open reading frame. In addition to miRNAs that could bind to the corresponding UTR, negative control pre-miRNA was transfected with the corresponding luciferase reporter plasmid for each gene analyzed. The luciferase activity was measured 24 hrs after transfection and the ratio of firefly∶renilla luciferase is expressed as percentage of control ± standard deviation shown. A statistically significant difference from control miRNA was calculated using one-way ANOVA and *p*-value of <0.003 are represented by * in the figure.

After the identification of the 63-set of miRNAs, we examined whether DRG-expressed genes can be targeted by these miRNAs. For our studies we chose *Cacna2d1*, *Scn11A* or *Nav1.9*, and *P2rx4*, each of which is well established in the neuropathic pain literature [10], [11], [12], [13], [14]. The mRNA data for these genes from our microarray expression profiling is shown in Table S1-A. A luciferase reporter assay was used to determine the effect of miRNA on gene function. HEK293 cells were co-transfected with synthetic miRNA and a luciferase reporter construct consisting of a 3′-UTR cloned downstream of the luciferase open reading frame. We selected miRNAs from our 63-set with high-scoring miRBase and TargetScan predictions towards the 3 selected target genes (Table S1-B). Our results show that for each luciferase reporter construct, at least one miRNA produced a significant suppression of activity (Figure 2). For *Scn11a* and *Cacna2d1*, the maximal suppression of gene expression was approximately 40%, and a reduction by approximately 50% was observed for *P2rx4* demonstrating that the 3′-UTRs of these genes can be targets for 63-set miRNAs, implying a potential role for these miRNAs in regulating DRG gene expression. Note that we achieved experimental validation in our luciferase reporter assay only for those miRNAs predicted through the TargetScan algorithm, while those miRNAs predicted through only mirBase showed no significant changes in luciferase activity (Table S1-B and Figure 2). This observation is in line with other reports that describe a better predictive power of TargetScan compared to other common algorithms [9].

### Prediction of miRNA targets and pathway analysis

To explore the likely biological consequences of SNL-mediated miRNA expression changes, we identified mRNA transcripts containing predicted target sites for these 63-set miRNAs. Since predicted sites of any one miRNA can number in the hundreds [15], [16], it was necessary to reduce these numbers to a manageable and biologically meaningful size. We used the TargetScan algorithm [15] for our predictions due in part to our earlier successes in predicting target gene modulation. Applying more stringent context score cutoffs increases the likelihood of observing biologically relevant effects when miRNAs are exogenously added *in vitro* [17]. For the predicted target sites of the 63 miRNAs, we applied a context score filter above the 60^th^ percentile, which reduced the number to 10,751 predicted target sites in 4899 rat genes.

**Figure 3:**
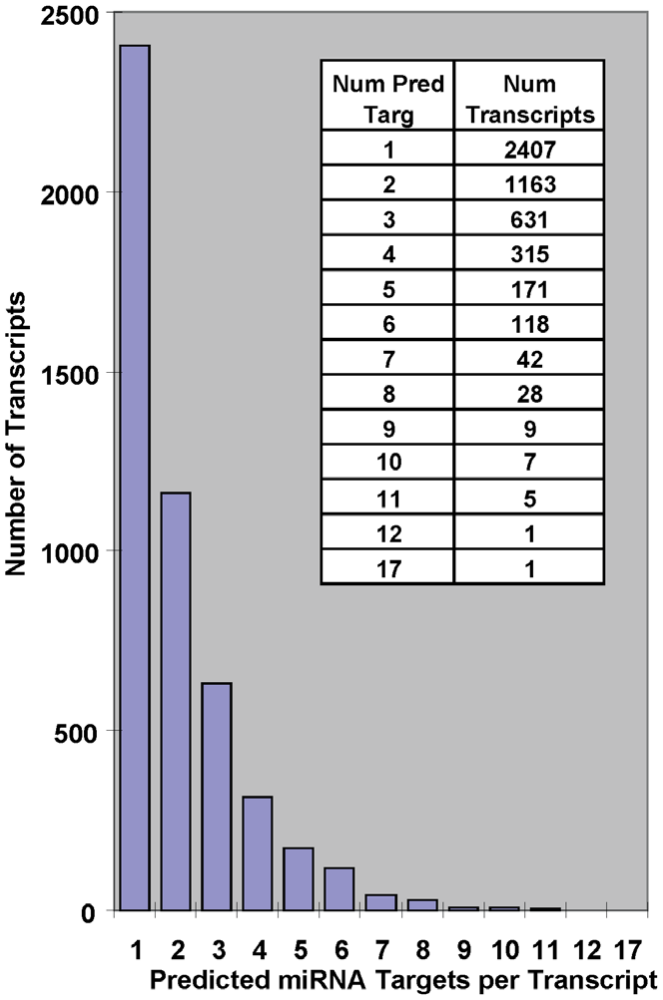
Distribution of predicted 63-set target sites in individual transcripts. The TargetScan algorithm was used for miRNA target prediction and there are hundreds of transcripts containing multiple predicted 63-set target sites. We increased the stringency for the predicted target sites of the 63 miRNAs by applying a context score filter above the 60th percentile. This reduced the number to 10,751 predicted target sites in 4899 rat genes.

Multiple miRNA target sites within a 3′-UTR suggest that miRNAs act together to orchestrate the modulation of mRNA translation [17], [18], [19], [20], [21]. Figure 3 shows that there are hundreds of transcripts containing multiple predicted 63-set target sites. Note that the number of 63-set target sites on a single transcript is not simply driven by 3′-UTR length (Table S2). Transcripts with few predicted 63-set target sites may be functionally diverse, while those with a larger number of sites may be more likely to be important to, and part of, a network of biologically relevant response genes regulated by these miRNAs in this pain model. To test this, we created 3 lists of target genes having a successively increasing number of predicted 63-set target sites (3 or more, 4 or more, and 5 or more which contained 1328, 697 and 382 genes, respectively) and then asked if requiring more 63-set target sites leads to an enrichment of gene functions relevant to this model, i.e. the nervous system.

**Figure 4:**
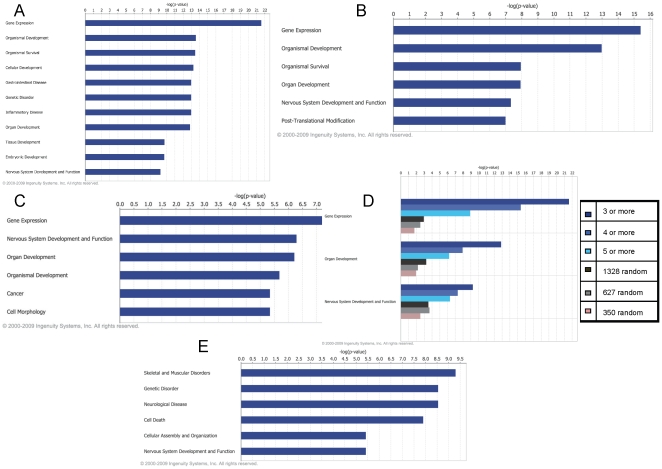
Ingenuity Pathway Core Analysis functions. Analysis of gene transcripts which had three-or-more (Panel A), four-or-more (Panel B) and five-or-more (Panel C), predicted 63-set target sites and a TargetScan context score above the 60^th^ percentile shown. The listed functions under the graphics are from the category called “Nervous System Development and Function.” The functions are sorted by *p*-value and only rows having 10 or more molecules are shown. Controls (Panel D) of randomly chosen gene sets of comparable sizes to the five-, four-, and three-or-more gene sets were also run through Ingenuity Pathway Core Analysis and appear in this Analysis Comparison. Only the top three broad categories are shown and they are sorted by the five-or-more order of functions. Overlap (Panel E) between the L4 ipsi- vs. contralateral mRNA transcripts and predicted mRNA targets of 63-set miRNA (60^th^ percentile) were run through Ingenuity Pathway Core Analysis. The top six functional categories are shown and the sub-categories under “Nervous System Development and Function” are listed, sorted by *p*-value and only rows having 10 or more molecules are shown.

These 3 sets of target genes were imported into the Ingenuity Pathway Analysis (IPA) tool to identify biological activities based upon the underlying literature derived annotation. Figure 4 (panels A to C) shows functions ranked by IPA's negative log *p*-value, signifying overrepresentation of genes relevant to the described function. Interestingly, with an increasing requirement for multiple target sites the tissue-specific listing “Nervous System Development and Function” rises through the ranking of functions, from 11^th^ to 2^nd^ position. This suggests that the TargetScan predicted gene targets and our filtering approach enriched for neurological events and functions. For a list of all genes with 3 or more predicted target sites, see Table S3.

Closer examination of the subcategories of “Nervous System Development and Function” shows neurogenesis and branching of neurites as the top functional categories (additional details are shown in the Supporting information for table listings under Figure 4 and Table S4). Randomly chosen gene lists of comparable sizes were analyzed in the IPA tool and demonstrate substantially lower negative log *p*-values in these same functional categories. (Panel D, Figure 4). An overlapping set of 153 transcripts emerges when the 1328 predicted 63-set target mRNAs (3 or more sites with a TargetScan context score of 60th percentile) were compared to the 1806 differentially expressed messenger RNA transcripts identified in a SNL-L4 ipsilateral versus contralateral comparison. These genes were subjected to IPA Core Analysis (direct interactions, only). The third and sixth ranking functions are “Neurological Disease” and “Nervous System Development and Function,” respectively, both with significant *p*-values. Consistent with the findings above, the top subfunctions under “Nervous System Development and Function” include the growth and development of neurites (Panel E, Figure 4).

We used SigPathway for differential expression studies at the miRNA target level. The bioinformatic prediction algorithm picTar [19] was used to map miRNAs to their target mRNA, which included transcripts that were shown to be significantly altered in a transcriptional profiling of the same RNA samples (data not shown; GEO accession number GSE24982). Targets for 51 of the 63 significantly changing miRNAs are predicted by picTar and 18 of these 51 are changing at the target mRNA level. However, there was no strict inverse correlation between miRNAs of our 63-set and their corresponding mRNA targets (data not shown).

### Text-mining of miRNA associations with gene function

Text mining of the existing miRNA literature [22] combined with the wealth of existing information on gene function was used to predict biological outcomes due to our observed miRNA changes. By text mining PubMed we could identify publications citing 61 of the 63-set miRNAs (97%), only miRs-409-5p and -572 were unpublished. Further mining of these miRNA-citing publications for relationships between 63-set miRNAs and human genes revealed 39 of the 63-set (60%) interacting with 153 human genes (Table S5). We found 6 of the 10-set miRNAs interacting with 27 human genes. Interestingly, many of the genes interacting with the 10-set miRNAs are transcription factors. To determine whether there is a functional relationship between the 63 miRNAs and pain, we used the National Library of Medicine's Medical Sub-heading (MeSh) biomedical ontology to identify pain-related terms from our text-mining of PubMed. By this method, only one miRNA (miR-10A) could be linked to pain, reflecting the lack of miRNA literature in the pain field [23]. It is to be noted that miRNA research is a rapidly expanding field and the list of genes and interactions are constantly evolving with an increase in publications.

To investigate what biological processes are associated with the 153 miRNA-target set, we used DAVID (http://david.abcc.ncifcrf.gov/) [24], [25] to look for enrichment of functions within the Biological Process section of the Gene Ontology (GO) [26]. DAVID clusters the list of enriched processes into functional groups based upon associated ontological terms. Our analysis showed that neurological processes were highly enriched for the 153 miRNA-target set genes (Table S6). DAVID identified several enriched clusters containing broad neurological terms with associated sub-categories that point to general neuronal remodeling processes, with recurring themes such as neurite/axon/dendrite growth, differentiation and regulation. Given the enrichment of neuronal remodeling processes observed in the results from DAVID, we text-mined PubMed to search for associations between these processes and the miRNA-target genes. We identified 30 miRNA-target genes with known roles in neurite or axon growth (Table S7) and curated the text-mined miR:gene:neurite phenotype associations to predict neurite growth outcomes in our model. The output from this analysis suggests that down-regulating members of the 63-set miRNAs may increase neurite outgrowth.

### siRNA knockdown of predicted target genes

We investigated the neurite outgrowth functionality encoded by presumptive target transcripts of 63-set miRNAs and were able to support some of our predictions using data from an unrelated siRNA screening project that analyzed genes by high content screening (HCS) for their ability to regulate neurite growth in differentiated Neuro2A cells (Figure S2). A gene whose knockdown by complementary siRNA inhibited growth, would be considered a neurite outgrowth promoting gene, and *vice versa*. We identified HCS data for 6 genes from our text mining predictions. We observed that 5 out of 6 of the siRNA inhibited genes affected neurite outgrowth in a manner that matched our predictions, that is, inhibition of the specific target gene reduced neurite growth (Table 2). This corroboration of several of our predictions leads us to hypothesize that under normal conditions, some of the miRNAs from the 63-set may act as repressors of neurite outgrowth. In our SNL-model this repression could be alleviated by the observed down-regulation of these miRNAs.

**Table 2:** Selected genes targeted by siRNAs during differentiation of Neuro2a cells.

Gene	Feature	*p*-Value			Percent change		
		siRNA1	siRNA2	siRNA3	siRNA1	siRNA2	siRNA3
*Akt3*	Neurite Avg Length	**9.9E-03**	**1.2E-05**	8.9E-01	*−0.10*	*−0.37*	−0.01
*Akt3*	Branch Point Avg Count	8.6E-01	**6.9E-06**	**1.6E-05**	−0.01	*−0.63*	*−0.28*
*Akt3*	Neurite Count	3.3E-01	**2.5E-05**	**5.9E-03**	−0.02	*−0.36*	*−0.09*
*Creb1*	Neurite Avg Length	**1.2E-02**	4.0E-01	**2.3E-04**	*−0.08*	*−0.05*	*−0.19*
*Creb1*	Branch Point Avg Count	**7.1E-04**	**5.0E-02**	**3.9E-05**	*−0.25*	*−0.10*	*−0.54*
*Creb1*	Neurite Count	**1.8E-04**	**4.2E-02**	**6.3E-04**	*−0.14*	0.04	*−0.14*
*Crk*	Neurite Avg Length	**7.5E-09**	**2.3E-07**	**3.4E-10**	*−0.45*	*−0.29*	*−0.29*
*Crk*	Branch Point Avg Count	**5.3E-07**	**6.5E-06**	**1.7E-05**	*−0.53*	*−0.23*	*−0.28*
*Crk*	Neurite Count	**1.1E-05**	**3.2E-03**	**1.2E-02**	*−0.21*	*−0.09*	*−0.05*
*Myc*	Neurite Avg Length	**3.6E-04**	**4.5E-04**	**8.5E-09**	**0.19**	**0.20**	**0.68**
*Myc*	Branch Point Avg Count	**2.4E-02**	**1.6E-05**	**1.8E-05**	**0.09**	**0.20**	**0.44**
*Myc*	Neurite Count	3.1E-01	**3.5E-07**	**7.9E-06**	−0.02	*−0.15*	*−0.12*
*Srf*	Neurite Avg Length	6.0E-01	**1.1E-05**	**2.2E-07**	0.03	*−0.17*	*−0.44*
*Srf*	Branch Point Avg Count	**8.0E-05**	**2.0E-08**	**1.4E-06**	*−0.17*	*−0.35*	*−0.42*
*Srf*	Neurite Count	1.9E-01	**2.2E-03**	7.8E-01	−0.03	*−0.07*	−0.01
*Tnf*	Neurite Avg Length	**3.4E-06**	**6.3E-05**	5.3E-01	*−0.49*	**0.29**	***0.03***
*Tnf*	Branch Point Avg Count	**6.6E-05**	9.9E-01	**3.0E-03**	*−0.68*	0.00	*−0.31*
*Tnf*	Neurite Count	**5.4E-08**	8.1E-01	**1.3E-06**	*−0.57*	−0.01	*−0.24*

Genes were each targeted by 3 independent siRNAs as indicated. The features of average neurite length and neurite count were determined and are shown here. *p*-values in comparison to non-targeting control siRNAs are reported. Statistically significant changes are highlighted in bold. Percent changes in the features in comparison to the same negative controls are also reported with increases highlighted in bold and decreases in italics (changes <.04 are not highlighted).

### Predicted Pathways from mRNA expression

**Figure 5:**
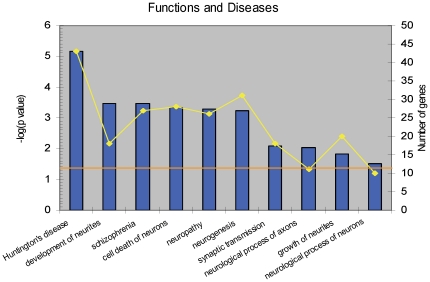
Functional categories perturbed in the SNL L4 DRGs. Functional processes related to Nervous System Development and Function with a minimum of 10 genes are indicated. The bars represent the negative (log *p*-value) and the orange data points indicate the number of genes for the given functional category. P-values indicate the probability that the observed association between the described functional categories and the SNL pain gene set is due to chance. The ratio indicates the percentage of genes in these functional categories that are found in the SNL pain gene set. The ratio thus indicates the strength of the association, and the *p*-value measures the statistical significance. The horizontal line indicates a *p*-value of 0.05.

Since we were also interested to see if pathways predicted to be regulated by miRNA changes are also implicated through their messenger-RNA changes we subjected SNL-regulated mRNA transcripts to Ingenuity Pathway Analysis. Functions and diseases (as defined in IPA) filtered for a nervous system context with a minimum of 10 genes per category and significantly perturbed in the SNL L4-specific DRG mRNA expression profiling dataset (FC>+/−1.5 & *p*-value<0.001) are shown in Figure 5. These belong to the top scoring “Neurological Disease” category (rank 1 with *p*-value range 5.73E-09 to 2.87E-02) and “Nervous System Development and Function” category (rank 22 with *p*-value range 3.36E-04 to 3.20E-02) relative to a total of 72 Ingenuity defined broad function and disease categories. Development and growth of neurites, neuropathy, and neuronal apoptosis are some of the dysregulated functional processes in the SNL L4 DRGs.

## Discussion

We report here miRNA changes in the SNL model of neuropathic pain, adding this to the growing list of conditions for which dysregulation of miRNA has been reported [27] (http://www.mir2disease.org/). We have demonstrated SNL induced miRNA changes in both L4 and L5 DRG, but the number of miRNAs affected, as well as the magnitude of the changes, were larger in L4 DRG. In the SNL model, tight ligation of the L5 spinal nerve results in ectopic activity not only in the injured L5 afferents, but in uninjured ipsilateral L4 afferents as well. Ectopic activity in both L4 and L5 suggests the possibility that the two populations of afferents are each capable of initiating and maintaining the behavioral changes resulting from nerve injury. Several studies support the hypothesis that these SNL-induced changes are initiated and maintained by activity originating from the injured afferents [28], [29]. The alternative or uninjured/intact afferent hypothesis suggests that changes in the uninjured and not the injured afferents may underlie the development and maintenance of neuropathic pain [30], [31], [32]. It is possible that, while the injured L5 DRG plays a crucial role in the early stages of injury, the uninjured L4 DRG may be playing a key role in the later stages [33].

After nerve injury, inflammatory mediators and aberrant neuronal activity set in motion transcriptional and posttranslational changes resulting in the induction and maintenance of neuropathic pain [34], [35], [36]. Molecular mechanisms contributing to peripheral and central sensitization in chronic pain have so far been studied only at the mRNA and protein levels. Pain model studies have measured mRNA levels by either looking at a targeted set of transcripts or through the use of global approaches such as microarray technology to study changes in neuropathic pain. In rat, 122 genes associated with the peripheral axotomy model for pain have been reported [5]. Prominent among the biological processes identified by pathway analysis in another study, performed two weeks after SNL, were functions such as neurite outgrowth and neuronal development, suggesting these effects to be among the primary consequences of SNL injury [37]. In a proteomic study [7], 67 proteins were found to be regulated seven days after SNL surgery. Proteins responsible for multiple cellular mechanisms such as structural and functional integrity of neurons were found to be regulated. One issue not addressed in these earlier studies is whether SNL-sensitive changes in mRNA or protein expression are preceded by changes in the expression of regulatory molecules such as miRNA.

Our miRNA profiling has identified a set of SNL-sensitive miRNAs and indicates that most of these were down-regulated following neuropathic injury. The resulting pattern was a clear separation of the ipsilateral L4 and L5 experimental groups from contralateral and sham-operated samples, which combine to compose a homogeneous group. This separation among the experimental groups was apparent before data normalization and was retained afterward. We employed a novel approach to miRNA normalization using a set of rank-preserved miRNAs with minimal fluctuation across all treatment groups. This method minimizes the potential bias that comes from the use of so-called housekeeping miRNAs or other small RNAs. Our observed changes in miRNA levels are greater than the SNL-associated mRNA changes [5], [6], a result which might suggest that the miRNA changes we observe propagate their effects on protein levels through translational regulation rather than through targeted mRNA degradation.

Our investigation of the biological consequences of SNL-induced miRNA changes combined experimental and bioinformatics approaches. By expressing the 3′-UTR sequence from three genes known to be expressed in DRG with certain SNL-regulated miRNAs, we demonstrated the potential for an impact of miRNA expression on target gene function. However, the large number of SNL-regulated miRNAs identified (hundreds) and their potential mRNA targets (thousands) prevented a comprehensive assessment of all possible miRNA/target interactions. To reduce the number of SNL-regulated miRNAs to a workable number, we imposed statistical filters (*p*<.01 and >2-fold change in expression), generating what we term the 63-set of SNL-regulated DRG miRNAs. Using published algorithms to predict target transcripts for these miRNAs, we identified putative target transcripts of the 63-set, which were in turn subjected to pathway analysis.

In light of the known mechanisms of miRNA influence on gene expression, it is not surprising that we did not observe a strict inverse correlation between the differentially regulated miRNAs and their predicted mRNA targets in our profiling studies. Our observation was a general down-regulation of miRNAs in our miRNA-profiling and a general upregulation of mRNAs in our transcriptional profiling studies. This pattern suggests that at least some of the down-regulated 63-set miRNAs could have acted through a transcriptional mechanism. Thus in addition to the classic switch interactions where miRNA induction results in the downregulation of preexisting mRNA targets, other types of interactions described as tuning and neutral are also possible [9], [38]. A large scale proteomics study performed to investigate the influence of specific miRNAs on protein levels revealed that hundreds of genes were directly, but modestly, repressed by individual miRNAs [39], [40]. Though many miRNA targets were affected by both repression of translation and mRNA degradation, a number of targets were predominantly regulated by translational repression [40]. Some were repressed without detectable changes in mRNA levels. In addition, transcription factors were some of the other major classes of genes that are perturbed by SNL in the L4 DRG in our study and could play an important role in gene regulation following SNL. It is also interesting to note that expression of several transcription factors are known to be regulated by miRNAs [41], [42]. In depth analysis of individual genes and the mode of action of miRNAs targeting them is needed for further elucidation of miRNA activity on specific target mRNAs in neuropathic pain.

The complexity of the regulatory network (i.e., many miRNAs affecting many target transcripts) makes it difficult to extract individual miRNA/target transcript interactions likely obscuring specific inverse correlations between the expression changes of miRNAs and their targets. In addition, the TLDA provides an incomplete survey of rat miRNAs. Any highly regulated miRNAs propagating their effects through target degradation, but which are not represented in the limited array, would be unavailable to our measuring of any existing correlation. Perhaps of greater importance, prediction of miRNA target sites is an evolving field. Any errors in prediction of miRNA target sites would undermine our ability to observe a correlation between miRNA and mRNA expression. A much more detailed follow-on study of individual genes and the miRNAs targeting them will be required to classify the mode of miRNA regulation in neuropathic pain. miRNA regulating target gene expression by altering the stability of the transcript and/or interference with the translational process adds another level of complexity that must be considered in dissecting the complex cascade of events that follow nerve injury during and after the development of chronic pain.

Rather than focusing on the individual miRNA-target interactions, pathway analysis and text mining methodologies were used to understand the biological functions and processes in pain. Much evidence exists that miRNAs can act together to repress the translation of their cognate mRNA targets [17], . Based on the expectation that the most profound downstream effects of SNL-mediated miRNA changes might be evident in the function of genes containing multiple miRNA targets, we performed pathway analysis on subsets of putative target mRNAs based on the number of predicted 63-set target sites per transcript. “Nervous System Development and Function” and its subcategory “Neurogenesis” are prominent in the pathway analysis output when such constrained input lists are considered. Comparison of genes common between the miRNA target site predictions (the 382 genes from the “5 or more list”) and SNL-regulated mRNAs (1806 transcripts found to significantly change in L4 DRG) showed an overlap of 46 genes. This overlapping gene set was also subjected to pathway analysis, with the result that Huntington's disease, neuropathy, and cell death of neurons are leading categories under “Neurological Diseases” and neurite growth and development are significant under “Nervous System Development and Function.” Text mining provided a complementary means of assessing function of the 63-set miRNAs and their putative target transcripts. By text mining, only one of the 63-set miRNAs has a published connection with pain, while many of the putative target transcripts (a set of 153 genes that contain 3 or more 63-set target sites and are significant mRNA change in L4 DRG) are associated with neuronal remodeling, a result that reinforces the results of pathway analysis.

The bioinformatic and pathway analysis approaches we pursued rely heavily on the current understanding of miRNA-mRNA interactions, something which is undergoing continual refinement within the miRNA research community. In contrast, text mining of literature-based information relies on published miRNA to mRNA target interactions, most of which have been confirmed experimentally. Interestingly, the biological functions associated with the set of genes identified by text mining as targets of our 63-set miRNAs bear a striking similarity to those functions identified by pathway analysis of the predicted target set. These separate approaches independently provide support for the idea that the identified miRNAs in our study play an important role in neuronal remodeling, as reflected by target genes functioning in neurite or axonal growth. Taken together, these findings suggest that neural remodeling activities are strongly associated with the set of transcripts that are among the most likely molecular targets of SNL-regulated miRNAs.

Neurons of the peripheral nervous system have the capacity to regenerate following injury. Regeneration (and the earlier degeneration) may have a significant role in the positive symptoms of pain resulting from nerve injury [45], [46]. Functional integration of peripheral and central nervous systems after nerve injury results in complex reorganization of circuits and could cause maladaptive changes resulting in pain, disesthesia, and hyperreflexia, all symptoms witnessed after spinal cord injury. Because SNL is a nerve injury model, it should not be surprising that, in our profiling of SNL-induced changes in DRG, miRNAs targeting genes involved in neurite outgrowth and axonal regeneration [47], [48] are downregulated. Extending our studies of miRNAs changes from the DRG to spinal cord would help us to better understand the larger role of these miRNAs in neuropathic pain. A recent study employing microarray analysis identified a set of miRNAs altered in spinal cord injury [49]. Eight of the miRNAs downregulated on day 7 were also downregulated in our studies. In a separate study, 12 miRNAs were reported to be enriched in mouse spinal cord. Of these twelve, eight are present in our 63-set with two being upregulated and 6 down regulated [50].

An experimental connection between putative 63-set targets and neurite outgrowth was derived from an independent siRNA survey of neurite outgrowth. In a high content screen of siRNAs for their ability to modulate neurite outgrowth in Neuro2A cells, six of the siRNAs screened were specific to members of the putative target gene set (the 153 genes mentioned above). In five of six cases, siRNA reduced neurite outgrowth, an outcome consistent with stimulation of neurite growth following downregulation of an miRNA targeting the same transcript. It should be pointed out that the HCS screen was performed independently of our profiling study and thus provided an unbiased confirmation of the function of these genes that were identified as putative miRNA targets. Further experiments are needed to confirm the hypothesis that miRNAs can play a role in suppressing neuronal remodeling.

In designing our studies, we chose to restrict our miRNA profiling to a single tissue and a single time point. DRG was chosen as the tissue for its well-documented role in pain signaling, and 4 weeks post-SNL was chosen as the time point because this is a time after the establishment of neuropathic pain. Consequently, the kinetics of miRNA regulation is not investigated in this study. Temporal regulation of mRNA expression in SNL is known [5], [6], [37] and the same is likely true of miRNA expression [23], [51]. A recent study, performed 2 weeks after SNL, focused on three miRNAs that are expressed as a cluster (miR-96, -182, and -183) in L5 DRG [52]. Significant downregulation was observed for miR-96 and miR-183 in L5 DRG. These two miRNAs showed altered expression in our study, but were excluded from our 63-set because the L4 comparison did not meet the *p*<0.01 cutoff we used in filtering the profiling output. In addition to differences in time point at which the experiments were performed, Aldrich et al used 4.5 S RNA as an endogenous control to normalize their data. We used a more stringent and novel approach to miRNA normalization using a set of rank-preserved miRNAs with minimal fluctuation across all treatment groups. This normalization method minimizes the potential bias that comes from the use of so-called housekeeping miRNAs or other small RNAs [53]. Future studies using tissues contributing to peripheral and central pain (DRG, spinal cord and brain regions), at multiple time points and in different pain models will be required for a full understanding of the role miRNAs play in the development and maintenance of chronic pain.

Our study provides an important first step toward understanding SNL-sensitivity of miRNA expression and the possible gene regulatory consequences of these expression changes. Additional studies in neuropathic pain validating the role of individual miRNAs, employing both cellular and *in vivo* miRNA delivery, should further elucidate the functional roles for these miRNAs in pain. Of equal benefit will be studies addressing the role of these SNL-regulated miRNAs in neuronal degeneration and regeneration. Of greatest importance, a better understanding of the molecular consequences of nerve injury could provide a first step toward development of novel therapies.

## Methods

### Spinal nerve ligation (SNL) model

Male Sprague–Dawley rats weighing 150–174 g were obtained from Harlan Laboratories (Indianapolis, IN) and were housed under controlled temperature and 12 h light/dark cycle for at least 6 days before any procedure. Food and water were provided *ad libitum*. SNL injury, or sham operation, was performed according to the procedure of Kim and Chung [4]. Briefly, under 2.5% isoflurane in O_2_, the dorsal vertebral column was exposed and the left L6 transverse process removed. The L5 spinal nerve was isolated and tightly ligated distal to the dorsal root ganglia with a 4-0 silk suture. The incisions were then closed. Sham control rats underwent the same operation and handling as the experimental animals, but without nerve ligation. Postoperatively, rats were housed individually under the same general conditions. All procedures were approved by Wyeth's Industrial Animal Care and Use Committee. Rats (SNL and sham) were tested for tactile sensitivity immediately prior to surgery (baseline) and before tissue extraction 4 weeks post-surgery to ensure SNL rats were hypersensitive. Tactile sensitivity was assessed by measuring each rat's paw withdrawal threshold in response to probing with von Frey monofilaments (Stoelting, Wood Dale, IL) according to the method of Chaplan et al. [54]. At week 4 after SNL or sham surgery, rats were deeply anesthetized with ketamine HCl. Bilateral L4 and L5 DRG's were excised, immediately frozen in dry ice and stored at −70°C.

### Total RNA isolation

Individual DRG tissue samples were homogenized using an Omni tissue homogenizer (TH) using Omni tip clear plastic homogenizing probes (Omni International, Marietta, GA). Total RNA was purified from individual DRGs using the mirVana™ miRNA Isolation Kit (Ambion, Austin, TX) following the manufacturer's instructions. RNA was quantified using a ND-1000 spectrophotometer (NanoDrop Technologies, Wilmington, DE).

### Description and overview of human miRNA TLDA panel

A Taqman Low Density Array (TLDA) Human miRNA Panel (rodent panel was not available at the time the experiments were performed) (Applied Biosystems, Foster City, CA), was used for measuring the expression levels of miRNAs expressed in rat DRGs according to the manufacturer's instructions. Bioinformatic miRNA sequence comparisons confirmed a large overlap in miRNA sequence between rat and human that allowed the use of a human miRNA TLDA for miRNA expression profiling of these rat tissues. Using strict standards, 57 of the rat miRNAs in the 63 set were found to be identical to the human sequence on the ABI array. Using only the human sequences on the ABI array, a total of 169 rat miRNAs were found to be identical to their human orthologs. Although this may seem low, almost twice the number of human miRNAs have been found compared to rat. Compared to mouse, only 64% of the rat miRNAs have been identified. Contributing reasons for the number discrepancy may be the incomplete rat genome (there are many gaps), and the attention given to human and mouse as model organisms for miRNA studies. Listing of miRNAs that are identical in human and rats are shown in Table S11.

### cDNA synthesis

Following the manufacturer's instructions, miRNAs were reverse transcribed from total RNA using the TaqMan MicroRNA Reverse Transcription Kit in conjunction with the Multiplex RT for TaqMan MicroRNA Assay pools (both Applied Biosystems, Foster City, CA); each total RNA sample (derived from one DRG) was divided into 8 fractions of 100 ng total RNA each and converted into a 8 multiplex pools of cDNA.

### TLDA TaqMan Real Time Assay

Following the manufacturers instructions for each multiplex RT reaction a TaqMan master mix was prepared using Taqman Universal PCR Master Mix (Applied Biosystems, Foster City, CA); a final volume of 100 µL/port (which supplies the master mix to 48 individual wells) was loaded into each of the 8 ports/TLDA card. TLDA cards were assayed on an ABI PRISM 7900 Sequence detector (Sequence Detector Software v2.2.2) using universal thermal cycling conditions of 50°C for 2 minutes, 95°C for 10 minutes, then 40 cycles of 95°C for 15 seconds and 60°C for 1 minute. We adapted the instrument default procedures of calculating relative quantification (RQ) to accommodate the multi-factorial design of this experiment. For consistent miRNA quantitation in all 40 samples, the threshold level for background detection in SDS software was manually set to 0.2.

### Statistical analysis design and rationale

A group of 10 animals was divided into 2 cohorts, (n = 5) one subjected to SNL injury and the other undergoing sham surgery. Bilateral L4 and L5 DRGs were harvested from each cohort, yielding a total of 40 samples for RNA isolation and TLDA processing. Within the SNL cohort, comparison of ipsilateral DRG (on the same side of the surgery) to the contralateral DRG provides an estimate of the combined “treatment” effect of SNL-induced pain effect (which is the effect of interest) plus any background effect that would be induced by surgery alone. This pure surgical effect can be considered an experimental artifact and therefore should be removed from the combined treatment effect. The estimation of this effect was achieved by the ipsilateral versus contralateral DRG comparison in the sham group.

### Data quality assessment and filtering

miRNA *C*_T_ values from all TLDA plates were collected and *C*_T_ distributions on each plate were inspected for data variability and sample anomalies. To reduce noise in the data for subsequent analysis, *C*_T_ readings flagged by instrument as “undetectable” (*C*_T_ = 40) were removed from the data set. The remaining 248 miRNAs were used for downstream analysis.

### Normalization

To investigate the issue of normalization, we first examined the endogenous controls on the TLDA card. Of the 3 controls only RNU6B was detected in the majority of DRG samples. Figure S1 shows the variation of *C*_T_ for RNU6B as well as the mean and median *C*_T_ calculated from all the detectable miRNAs on each TLDA plate. As shown in the figure, RNU6B closely follows the variation of mean and median plate *C*_T_ across the sample range which is driven by biological and experimental conditions. Because of lack of duplication wells for RNU6B located in an edge column, it is difficult to determine whether the *C*_T_ variation in RNU6B across the samples was due to plate-to-plate variation or random noise alone. Normalizing by plate mean or median *C*_T_ will result in “cancelation” of much of the biological variations. We sought an alternative normalization approach, based on the idea of choosing a minimal rank difference or rank-invariant miRNA set from within the data, a strategy commonly employed in microarray analysis [55]. Specifically, we identified a set of 11 detectable miRNAs on the TLDA card to serve as a normalization reference. The expression ranking of these miRNAs was minimally perturbed across the 40 samples (Figure S1). To normalize data, we first calculated the mean *C*_T_ values of all 11 miRNAs to derive a single reference profile as normalization baseline (plotted pink in Figure S1). Then, we subtracted the mean control miRNA *C*_T_ from the raw *C*_T_ of each miRNA on the same TLDA plate. These normalized expression had the same unit as Δ*C*_T_, and were used subsequently for differential expression analysis (Table S8).

### Statistical analysis

Because DRGs from both sides of L4 and L5 are from the same animals, a paired t-test was performed per miRNA to compare the ipsi- and contralateral DRGs within each segment in the SNL cohort, followed by false discovery rate (FDR) adjustment for multiple testing [55]. The same analysis procedures were also performed in the sham cohort. Signed fold changes were calculated using the standard formula, 2^−ΔΔ*C*^_T_, to convert the ΔΔ *C*_T_ obtained from the paired t-test into a linear scale, followed by taking the negative reciprocal of the fractional fold change. As described in the statistical design section, to obtain the SNL-specific pain effect, the treatment effect from the SNL cohort analysis needs be adjusted by the surgery effect estimated from the sham cohort. To perform the adjustment, we identified miRNAs with significant (FDR<5%) changes in expression in the sham comparison, and subtracted the fold change in the sham segment from the fold change estimated for the corresponding SNL segment. This procedure resulted in 4 (or 5) miRNAs whose fold change in the L4 (L5 respectively) segment was adjusted and with small magnitudes, suggesting overall surgery effect is largely negligible.

### Prediction of miRNA targets

The sequences of miRNAs were obtained from miRBase. Predicted miRNA targets and their associated genes were obtained from TargetScan and downloaded from the targetscan.org website. Sequences from the TLDA card were obtained directly from Applied Biosystems. Conserved predicted miRNA target sites identified with the TargetScan algorithm have context scores based on the location within the 3′-UTR, local AU composition at that location, and base-pairing at the 3 prime (3P) end of the miRNA. These scores were used to filter targets above the 60^th^ percentile.

For TargetScan data mining purposes, the 63-set list of miRNA names was modified to accommodate the differences in nomenclature between the ABI TLDA platform used to capture miRNAs and the TargetScan database of predicted target sites. For example, the miRNA designated as miR-10a by ABI is called miR-10a-5p by TargetScan. In addition to nomenclature issues, TargetScan groups paralogous miRNAs as having the same predicted target sites. For example, miR-30a and miR-30d have the same predicted target sites, so only miR-30a was used in our analysis to avoid duplication of data. A complete list of naming differences appears in the Table S9.

Gene symbols of transcripts with predicted target sites were used to obtain the annotation from the Ingenuity Pathway Analysis tool. Lists of these symbols were uploaded into the tool and Core Analysis was run looking at direct relationships only. Functions were examined and ranked by *p*-value.

### mRNA expression profiling, data normalization and filtering

Affymetrix rat genome GeneChips 430 2.0 were used for mRNA profiling and data was normalized using the MAS 5 algorithm. GeneData Expressionist enterprise system (http://www.genedata.com/products/expressionist.html) was used for statistical analyses. A *p*-value of <0.001 (FDR<1.5%) was used as a cutoff after a t-test analysis to identify probesets that were differentially expressed in the ipsi vs. contralateral L4 DRGs. FDR calculation was performed according to the procedures of Benjamini and Hochberg [56]. For the sham group 2101 probesets and for the SNL group 2239 probesets were identified. Of these differentially expressed probesets, 1806 probesets were specific to the SNL L4 DRGs.

The accession number for data deposited in Gene Expression Omnibus (GEO) is GSE24982.

### mRNA pathway analysis

Pathway analysis was performed using Ingenuity Pathways Analysis (IPA) (Ingenuity Systems, Redwood City, CA) and MetaCore (GeneGo; www.genego.com). A fold change of 1.5 was imposed on the 1806 probesets that are differentially expressed and specific to the SNL L4 DRGs. Transcription factor networks were built in MetaCore. Networks around known miRNA targets were built in IPA and MetaCore and significance of various GO categories and functional processes was analyzed for these miRNA-target networks. Interactome analysis was done in MetaCore.

### Identifying miRNA morphological variants

The most common miRNA morphological variants were identified following term-frequency analysis of a training set of full-text miRNA review articles. Curated regular expression patterns were developed to identify as many miRNAs as possible. Accuracy of regular expressions was tested by querying a download of annotation data from miRBase (http://microrna.sanger.ac.uk/sequences/ftp.shtml). Once acceptable accuracy and recall were achieved, regular expression patterns were imported into I2E for text-mining.

### Text-mining

All text-mining was performed using the I2E indexing/semantic search tool (Linguamatics, UK). Experimental literature corpuses used for text-mining were PubMed (NLM) and a set of approximately 3000 miRNA-citing full-text journal articles. Sentences containing miRNAs were identified using curated regular expression patterns. Semantic relationships were retrieved using the I2E's linguistic rules in combination with subject-specific dictionaries and ontologies. Dictionaries to identify human genes were derived from Entrez Gene (ftp://ftp.ncbi.nlm.nih.gov/gene/). Cellular processes were identified using the MeSH ontology (http://www.nlm.nih.gov/mesh/).

### Luciferase reporter gene assay

Plasmids for biological validation of miRNAs were constructed as follows. The 535 bp 3′-UTR of *Scn11a* (*Nav1.9*) (accession number NM_019265), 389 bp 3′-UTR of *Cacna2d1* (accession number NM_012919) and 710 bp 3′-UTR of *P2rx4* (accession number NM_031594) were synthesized with *Pme*1 and *Xho*1 restriction enzymes sites at the 5′ and 3′ ends respectively. The UTR fragments were then cloned into the pmirGLO Dual-Luciferase miRNA target expression vector (Promega, Madison, WI) and sequenced for confirmation. Lipofectamine 2000 mediated transfections of HEK293 cells in suspension were performed using 1 µg plasmid DNA and 100 nM of individual premiRNA. Fifty µl of cells (20,000cells/well) were plated in 96 well tissue-culture treated polystyrene black clear flat-bottom assay plates (BD Biosciences, San Jose, CA). Plates were maintained at 37°C in a humidified atmosphere containing 5% CO_2_. The functional assay for measuring firefly and renilla luciferase activity was done 24 hours after transfection according to manufacturer's protocol.

### siRNA screening of predicted target genes

Undifferentiated mouse Neuro2A cells (N2A) were maintained by regular passage in a growth medium consisting of Minimal Essential Medium with 10% FBS, 0.1 mM non-essential amino acids, 2 mM GlutaMax-1, and 1 mM sodium pyruvate (all from Invitrogen, Carlsbad, CA). Cells were transfected with custom designed siRNAs (Ambion, Austin, TX) via a reverse transfection procedure with Optifect (Invitrogen) following the manufacturer's recommended procedure. Briefly, 2 µl of each siRNA at a concentration of 1 µM was spotted into four independent wells of a 384-well dish and stored at −80°C until use. Three independent siRNAs per gene were spotted giving a total of twelve independent wells per gene per plate. N2A cells were detached from growth plates using trypsin and then resuspended in Opti-MEM I medium (Invitrogen) at a concentration of 50 cells/µl. Fifty nanograms of an EGFP expression plasmid (Clontech, Mountain View, CA) along with 0.5 µl of Optifect were then added per 20 µl of resuspended cells and plated (final 1000 cells/well). Cells were maintained overnight and the next day, switched to a differentiation medium consisting of growth medium with reduced FBS (2% final) and 8 µM retinoic acid (Sigma, St Louis, MO). Cells were maintained in this medium for four days then fixed with 4% paraformaldehyde. After fixation cells were labeled with rabbit anti-GFP (Novus, Littleton, CO) to detect transfected cells and mouse monoclonal anti-TuJ1 (Covance, Emeryville, CA) to detect differentiated cells. Anti-rabbit and anti-mouse secondary antibodies conjugated respectively with AF488 or AF594 (Invitrogen) were used for visualization. Nuclei were counterstained with Hoechst33258. Images of stained cells were captured using a Cellomics ArrayScan HCS Reader (Thermo Fisher Scientific, Pittsburgh, PA). Each well was fully scanned in three excitation/emission channels; 475/535 (green), 535/590 (red), 365/535 (blue). To be considered for analysis a minimum of 200 surviving cells per well was set as a lower limit. Images were processed using the Neuronal Profiling V.2 software application (Thermo Fisher) to quantify cell shape and process size parameters of cells staining positive in the red channel (TuJ1+). Images were gated such that only transfected (green) cells were considered in the analysis. The assay was repeated three times and a percent difference from the average of negative control siRNA transfected cells was calculated for each reported parameter. Negative control siRNAs (RISC-free siRNA #4390843 (Ambion) and NegsiRNA #1022076 (Qiagen, Valencia, CA) were also spotted into four independent wells each. siRNAs sequences are shown in Table S10.

The data analysis workflow was executed as follows. A set of 48 cytological parameters describing the morphological features of cells (e.g. cell body, nucleus, neurites) were extracted from the Cellomics database. These features were summarized at the well level by the Cellomics image analysis software Neuronal Profiling. After data quality inspection, plate-level normalization was performed using the control wells as the baseline. Normalized feature data were log2-transformed and the Student t-test was applied to each feature to compare siRNA transfection readout with the negative control. The test procedure was iterated for each siRNA (3 per gene) for all genes. “Gene hits” were identified for further evaluation based on statistical significance of knockdown effect (*p*-value<0.01 in at least 2 siRNAs per gene) plus relevant biological criterion such as percent difference above certain threshold (>25% in at least 2 siRNAs per gene).

## Supporting Information

Figure S1 **Assessment of** ***C*** **_T_ distribution of various controls for normalization.** Group-specific mean and median *C*_T_ for RNU6B (endogenous control) show a parallel trend of variation as the plate-specific mean and median *C*_T_ of the set of 248 detectable miRNAs (excluding RNU6B). This suggests that experimental variation may have been confounded with the biological variation. On average, the set of 11 rank-preserving (see text) miRNAs shows a reduced variation and was chosen as the normalization reference.
(TIFF)

Figure S2 **Example images of differentiated Neuro2a cells.** Cells transfected with negative control non-targeting siRNA (B, D) or siRNA targeting Crk. Green cells in C and D have taken up a co-transfected GFP plasmid and therefore indicate siRNA transfected cells. Note the neuritis protruding from green cells in D and the diminished size protruding from green cells in C. A and B show the same respective images of C and D with cell traces applied.
(TIFF)

Table S1 **A. Transcriptional profiling (mRNA) data for** ***P2rx4*** **,** ***Cacna2d1*** **and** ***Nav1.9*** **.** Each t-test comparison is between the ipsilateral versus the contralateral side (uncorrected p-values are shown). *Cacna2d1* is upregulated, *Nav1.9* (*Scn11a*) shows significant downregulation only in the SNL L5 DRG and *P2rx4* is not regulated significantly. **B. miRNA prediction using TargetScan and mirBase for** ***Cacna2d1*** **,** ***Nav1.9*** **and** ***P2rx4*** **.** Length of the 3′-UTR for these genes and fold change of the miRNAs in L4 and L5 DRG are also shown.
(DOC)

Table S2 **A sampling of the lengths of 3′-UTRs used by TargetScan in miRNA target prediction.** Two sets of sequences appear: those predicted to have 10 or more (N = 14) 63-set target sites and 14 randomly chosen transcripts predicted to have only one 63-set target site. The rows are sorted by 3′-UTR length and the unit of length is nucleotides.
(DOC)

Table S3 **Rat transcripts possessing 63-set miRNA target sites predicted by TargetScan.** Predictions have TargetScan context scores above the 60th percentile. The rows are sorted by the number of predicted 63-set miRNA target sites per transcript. The multiple miRNA target sites listed for a gene are ordered by decreasing TargetScan context score. Gene annotation provided by the Ingenuity Pathway Analysis tool.
(XLS)

Table S4 **Ingenuity Pathway Core Analysis.** Analysis of functions of 1328 genes with three or more predicted 63-set target sites and a TargetScan context score above the 60^th^ percentile. The listed functions are subcategories under the broader category called “Nervous System Development and Function.” The rows are sorted by *p*-value and lists with P-values of E-04 and lower are shown.
(DOC)

Table S5 **Text mining of miRNAs.** 39 microRNAs (of the 63-set) and their literature associated human target genes (Entrez gene symbols) uncovered from a systematic mining of PubMed. Verb-group triplets were identified using regular expressions and a gene thesaurus using the I2E semantic search tool. All entries in bold represent the 10-set.
(DOC)

Table S6 **Partial results from GSEA of 153 miRNA-regulated genes against GO Biological Processes (DAVID).**
(DOC)

Table S7 **Neurite or axonal phenotypes regulated by the 63-set associated gene.**
(DOC)

Table S8 **Complete list of rank normalized** ***C*** **_T_ values for the 63-set miRNAs for all samples.**
(XLS)

Table S9 **microRNA nomenclature.**
(DOC)

Table S10 **siRNA sequences used in screening of predicted target genes.**
(XLS)

Table S11 **Comparison of rat and human miRNAs.**
(XLS)

## References

[1] O'Connor AB, Dworkin RH (2009). Treatment of neuropathic pain: an overview of recent guidelines.. Am J Med.

[2] Seal RP, Wang X, Guan Y, Raja SN, Woodbury CJ (2009). Injury-induced mechanical hypersensitivity requires C-low threshold mechanoreceptors.. Nature.

[3] Costigan M, Scholz J, Woolf CJ (2009). Neuropathic pain: a maladaptive response of the nervous system to damage.. Annu Rev Neurosci.

[4] Kim SH, Chung JM (1992). An experimental model for peripheral neuropathy produced by segmental spinal nerve ligation in the rat.. Pain.

[5] Xiao HS, Huang QH, Zhang FX, Bao L, Lu YJ (2002). Identification of gene expression profile of dorsal root ganglion in the rat peripheral axotomy model of neuropathic pain.. Proc Natl Acad Sci U S A.

[6] Costigan M, Befort K, Karchewski L, Griffin RS, D'Urso D (2002). Replicate high-density rat genome oligonucleotide microarrays reveal hundreds of regulated genes in the dorsal root ganglion after peripheral nerve injury.. BMC Neurosci.

[7] Komori N, Takemori N, Kim HK, Singh A, Hwang SH (2007). Proteomics study of neuropathic and nonneuropathic dorsal root ganglia: altered protein regulation following segmental spinal nerve ligation injury.. Physiol Genomics.

[8] Erson AE, Petty EM (2008). MicroRNAs in development and disease.. Clin Genet.

[9] Bartel DP (2009). MicroRNAs: target recognition and regulatory functions.. Cell.

[10] Luo ZD, Chaplan SR, Higuera ES, Sorkin LS, Stauderman KA (2001). Upregulation of dorsal root ganglion (alpha)2(delta) calcium channel subunit and its correlation with allodynia in spinal nerve-injured rats.. J Neurosci.

[11] Taylor CP (2009). Mechanisms of analgesia by gabapentin and pregabalin–calcium channel alpha2-delta [Cavalpha2-delta] ligands.. Pain.

[12] Ulmann L, Hatcher JP, Hughes JP, Chaumont S, Green PJ (2008). Up-regulation of P2X4 receptors in spinal microglia after peripheral nerve injury mediates BDNF release and neuropathic pain.. J Neurosci.

[13] Tsuda M, Shigemoto-Mogami Y, Koizumi S, Mizokoshi A, Kohsaka S (2003). P2X4 receptors induced in spinal microglia gate tactile allodynia after nerve injury.. Nature.

[14] Cummins TR, Sheets PL, Waxman SG (2007). The roles of sodium channels in nociception: Implications for mechanisms of pain.. Pain.

[15] Lewis BP, Burge CB, Bartel DP (2005). Conserved seed pairing, often flanked by adenosines, indicates that thousands of human genes are microRNA targets.. Cell.

[16] Griffiths-Jones S, Saini HK, van Dongen S, Enright AJ (2008). miRBase: tools for microRNA genomics.. Nucleic Acids Res.

[17] Grimson A, Farh KK, Johnston WK, Garrett-Engele P, Lim LP (2007). MicroRNA targeting specificity in mammals: determinants beyond seed pairing.. Mol Cell.

[18] Doench JG, Sharp PA (2004). Specificity of microRNA target selection in translational repression.. Genes Dev.

[19] Krek A, Grun D, Poy MN, Wolf R, Rosenberg L (2005). Combinatorial microRNA target predictions.. Nat Genet.

[20] Lai M, Macleod M (2005). MicroRNA–taking regulation of protein synthesis to another level.. Cerebrovasc Dis.

[21] Saetrom P, Heale BS, Snove O, Aagaard L, Alluin J (2007). Distance constraints between microRNA target sites dictate efficacy and cooperativity.. Nucleic Acids Res.

[22] Murray BS, Choe SE, Woods M, Ryan TE, Liu W (2010). An in silico analysis of microRNAs: mining the miRNAome.. Mol Biosyst.

[23] Bai G, Ambalavanar R, Wei D, Dessem D (2007). Downregulation of selective microRNAs in trigeminal ganglion neurons following inflammatory muscle pain.. Mol Pain.

[24] Huang da W, Sherman BT, Lempicki RA (2009). Systematic and integrative analysis of large gene lists using DAVID bioinformatics resources.. Nat Protoc.

[25] Dennis G, Sherman BT, Hosack DA, Yang J, Gao W (2003). DAVID: Database for Annotation, Visualization, and Integrated Discovery.. Genome Biol.

[26] Ashburner M, Ball CA, Blake JA, Botstein D, Butler H (2000). Gene ontology: tool for the unification of biology. The Gene Ontology Consortium.. Nat Genet.

[27] Jiang Q, Wang Y, Hao Y, Juan L, Teng M (2009). miR2Disease: a manually curated database for microRNA deregulation in human disease.. Nucleic Acids Res.

[28] Sheen K, Chung JM (1993). Signs of neuropathic pain depend on signals from injured nerve fibers in a rat model.. Brain Res.

[29] Yoon YW, Na HS, Chung JM (1996). Contributions of injured and intact afferents to neuropathic pain in an experimental rat model.. Pain.

[30] Gold MS (2000). Spinal nerve ligation: what to blame for the pain and why.. Pain.

[31] Ali Z, Ringkamp M, Hartke TV, Chien HF, Flavahan NA (1999). Uninjured C-fiber nociceptors develop spontaneous activity and alpha-adrenergic sensitivity following L6 spinal nerve ligation in monkey.. J Neurophysiol.

[32] Li Y, Dorsi MJ, Meyer RA, Belzberg AJ (2000). Mechanical hyperalgesia after an L5 spinal nerve lesion in the rat is not dependent on input from injured nerve fibers.. Pain.

[33] Jang JH, Kim KH, Nam TS, Lee WT, Park KA (2007). The role of uninjured C-afferents and injured afferents in the generation of mechanical hypersensitivity after partial peripheral nerve injury in the rat.. Exp Neurol.

[34] Schmitt AB, Breuer S, Liman J, Buss A, Schlangen C (2003). Identification of regeneration-associated genes after central and peripheral nerve injury in the adult rat.. BMC Neurosci.

[35] Ji RR, Strichartz G (2004). Cell signaling and the genesis of neuropathic pain.. Sci STKE.

[36] Vanderluit JL, McPhail LT, Fernandes KJ, McBride CB, Huguenot C (2000). Caspase-3 is activated following axotomy of neonatal facial motoneurons and caspase-3 gene deletion delays axotomy-induced cell death in rodents.. Eur J Neurosci.

[37] Wang H, Sun H, Della Penna K, Benz RJ, Xu J (2002). Chronic neuropathic pain is accompanied by global changes in gene expression and shares pathobiology with neurodegenerative diseases.. Neuroscience.

[38] Fabian MR, Sonenberg N, Filipowicz W (2010). Regulation of mRNA translation and stability by microRNAs.. Annu Rev Biochem.

[39] Baek D, Villen J, Shin C, Camargo FD, Gygi SP (2008). The impact of microRNAs on protein output.. Nature.

[40] Selbach M, Schwanhausser B, Thierfelder N, Fang Z, Khanin R (2008). Widespread changes in protein synthesis induced by microRNAs.. Nature.

[41] Yun SJ, Byun K, Bhin J, Oh JH, Nhung le TH (2010). Transcriptional regulatory networks associated with self-renewal and differentiation of neural stem cells.. J Cell Physiol.

[42] Martino S, di Girolamo I, Orlacchio A, Datti A (2009). MicroRNA implications across neurodevelopment and neuropathology.. J Biomed Biotechnol.

[43] Lai EC, Tam B, Rubin GM (2005). Pervasive regulation of Drosophila Notch target genes by GY-box-, Brd-box-, and K-box-class microRNAs.. Genes Dev.

[44] Lai EC (2005). miRNAs: whys and wherefores of miRNA-mediated regulation.. Curr Biol.

[45] Campbell JN (2001). Nerve lesions and the generation of pain.. Muscle Nerve.

[46] Navarro X, Vivo M, Valero-Cabre A (2007). Neural plasticity after peripheral nerve injury and regeneration.. Prog Neurobiol.

[47] Vo N, Klein ME, Varlamova O, Keller DM, Yamamoto T (2005). A cAMP-response element binding protein-induced microRNA regulates neuronal morphogenesis.. Proc Natl Acad Sci U S A.

[48] Wayman GA, Davare M, Ando H, Fortin D, Varlamova O (2008). An activity-regulated microRNA controls dendritic plasticity by down-regulating p250GAP.. Proc Natl Acad Sci U S A.

[49] Liu NK, Wang XF, Lu QB, Xu XM (2009). Altered microRNA expression following traumatic spinal cord injury.. Exp Neurol.

[50] Bak M, Silahtaroglu A, Moller M, Christensen M, Rath MF (2008). MicroRNA expression in the adult mouse central nervous system.. RNA.

[51] Sonkoly E, Wei T, Janson PC, Saaf A, Lundeberg L (2007). MicroRNAs: novel regulators involved in the pathogenesis of Psoriasis?. PLoS One.

[52] Aldrich BT, Frakes EP, Kasuya J, Hammond DL, Kitamoto T (2009). Changes in expression of sensory organ-specific microRNAs in rat dorsal root ganglia in association with mechanical hypersensitivity induced by spinal nerve ligation.. Neuroscience.

[53] Mestdagh P, Van Vlierberghe P, De Weer A, Muth D, Westermann F (2009). A novel and universal method for microRNA RT-qPCR data normalization.. Genome Biol.

[54] Chaplan SR, Bach FW, Pogrel JW, Chung JM, Yaksh TL (1994). Quantitative assessment of tactile allodynia in the rat paw.. J Neurosci Methods.

[55] Li C, Hung Wong W (2001). Model-based analysis of oligonucleotide arrays: model validation, design issues and standard error application.. Genome Biol.

[56] Benjamini Y, Hochberg Y (1995). Controlling the false discovery rate: a practical and powerful approach to multiple testing.. Journal of the Royal Statistical Society Series B.

