# Supplementary figures and images for: Dynamic Changes in the MicroRNA Expression Profile Reveal Multiple Regulatory Mechanisms in the Spinal Nerve Ligation Model of Neuropathic Pain
Source: PLoS One. 2011 Mar 14;6(3):e17670. doi: 10.1371/journal.pone.0017670 (PMC3056716; doi:10.1371/journal.pone.0017670)

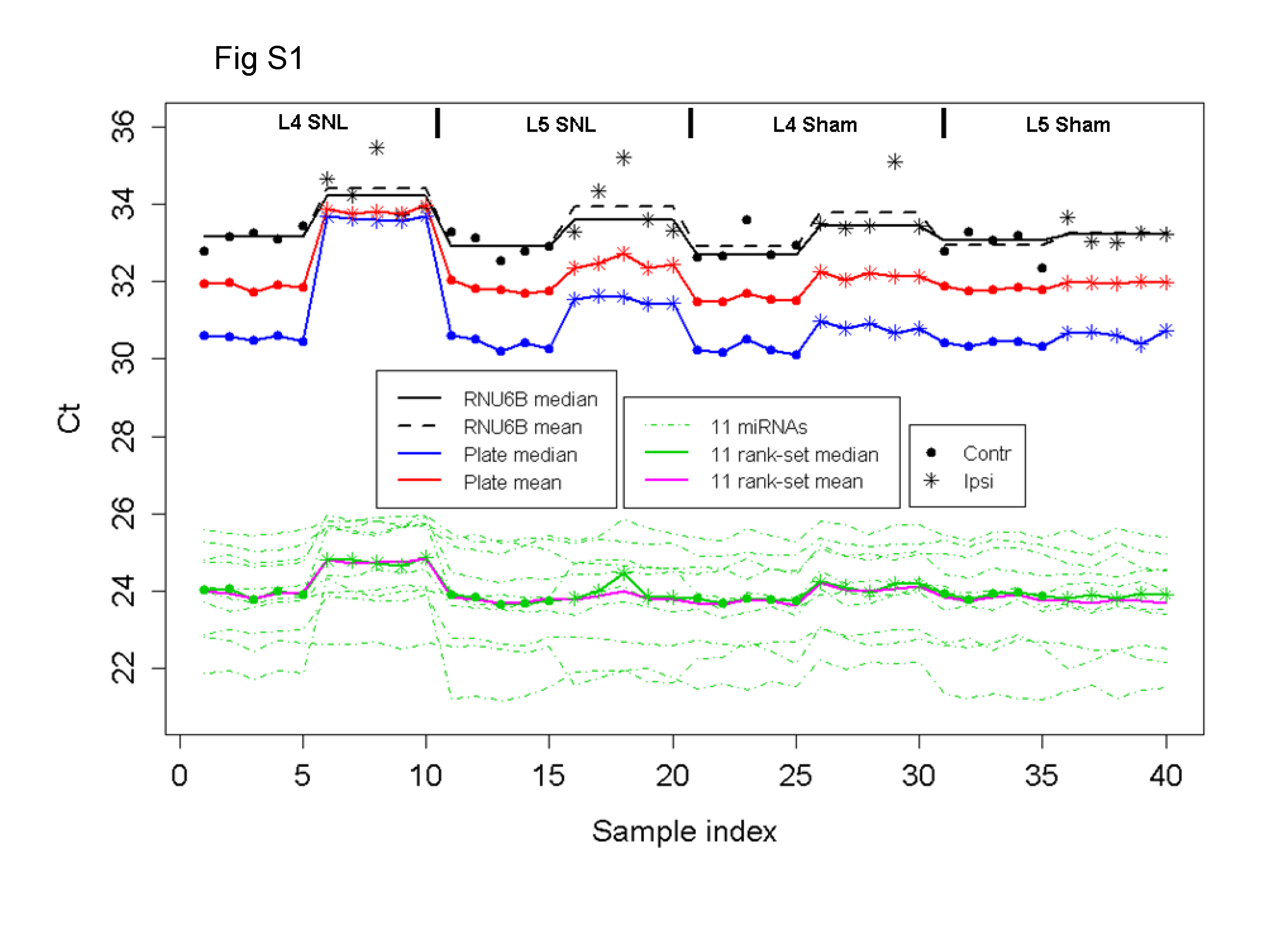

Supplement: Figure S1 — Assessment of C T distribution of various controls for normalization. Group-specific mean and median C T for RNU6B (endogenous control) show a parallel trend of variation as the plate-specific mean and median C T of the set of 248 detectable miRNAs (excluding RNU6B). This suggests that experimental variation may have been confounded with the biological variation. On average, the set of 11 rank-preserving (see text) miRNAs shows a reduced variation and was chosen as the normalization reference. (TIFF) [file pone.0017670.s001.tiff]

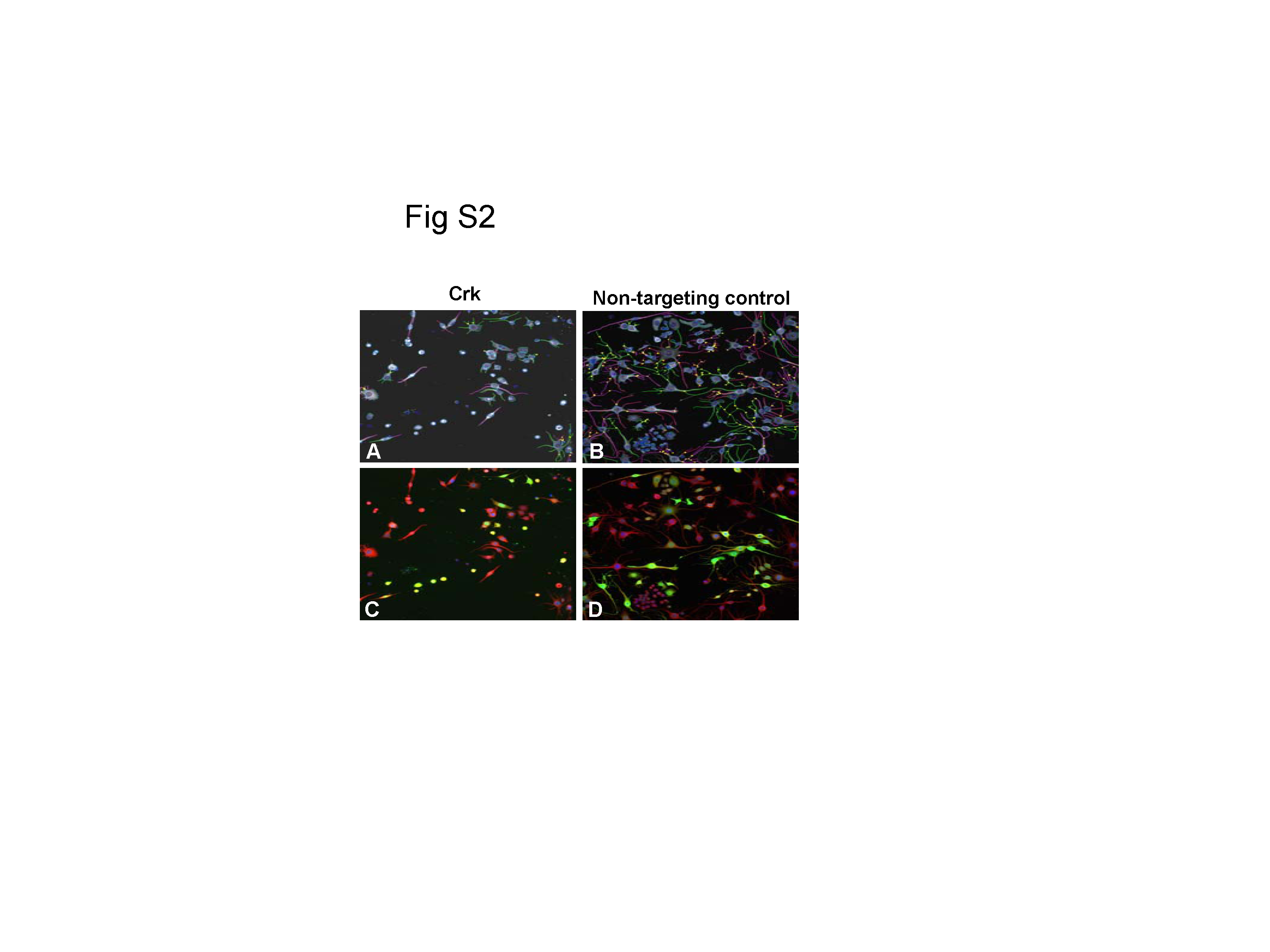

Supplement: Figure S2 — Example images of differentiated Neuro2a cells. Cells transfected with negative control non-targeting siRNA (B, D) or siRNA targeting Crk. Green cells in C and D have taken up a co-transfected GFP plasmid and therefore indicate siRNA transfected cells. Note the neuritis protruding from green cells in D and the diminished size protruding from green cells in C. A and B show the same respective images of C and D with cell traces applied. (TIFF) [file pone.0017670.s002.tiff]
