# Supplementary material for: Dynamic Changes in the MicroRNA Expression Profile Reveal Multiple Regulatory Mechanisms in the Spinal Nerve Ligation Model of Neuropathic Pain
Source: PLoS One. 2011 Mar 14;6(3):e17670. doi: 10.1371/journal.pone.0017670 (PMC3056716; doi:10.1371/journal.pone.0017670)
Supplement: Table S1 — A. Transcriptional profiling (mRNA) data for P2rx4 , Cacna2d1 and Nav1.9 . Each t-test comparison is between the ipsilateral versus the contralateral side (uncorrected p-values are shown). Cacna2d1 is upregulated, Nav1.9 (Scn11a) shows significant downregulation only in the SNL L5 DRG and P2rx4 is not regulated significantly. B. miRNA prediction using TargetScan and mirBase for Cacna2d1 , Nav1.9 and P2rx4 . Length of the 3′-UTR for these genes and fold change of the miRNAs in L4 and L5 DRG are also shown. (DOC) [file pone.0017670.s003.doc]

**Table S1 – A. Transcriptional profiling (mRNA) data for *P2rx4*, *Cacna2d1* and *Nav1.9*.** Each T-test comparison is between the ipsilateral versus the contralateral side (uncorrected p-values are shown). *Cacna2d1* is upregulated, *Nav1.9* (*Scn11a*) shows significant downregulation only in the SNL L5 DRG and *P2rx4* is not regulated significantly.

| Affy Qualifier | 1369649_at | 1368768_at | 1369743_a_at |
| --- | --- | --- | --- |
| Gene | ***Cacna2d1*** | ***Nav1.9 (Scn11a)*** | ***P2rx4*** |
| T-Test_SNL_L4: P-Value | 4.98E-06 | 0.3831449 | 0.1515283 |
| T-Test_SNL_L4: Ratio of Means | 0.4954657 | 1.035349 | 0.7957372 |
| T-Test_SNL_L5: P-Value | 8.32E-09 | 1.77E-10 | 0.165434 |
| T-Test_SNL_L5: Ratio of Means | 2.332474 | 0.1090504 | 1.169077 |
| T-Test_ham_L4: P-Value | 0.000926626 | 0.7935182 | 0.1554247 |
| T-Test_Sham_L4: Ratio of Means | 0.6307228 | 0.9840932 | 0.8899584 |
| T-Test_Sham_L5: P-Value | 0.007843331 | 0.6453385 | 0.03299367 |
| T-Test_Sham_L5: Ratio of Means | 1.135583 | 0.9821773 | 1.274943 |

**Table S1 – B. miRNA prediction using TargetScan and mirBase for *Cacna2d1*, *Nav1.9* and *P2rx4*.** Length of the 3’-UTR for these genes and fold change of the miRNAs in L4 and L5 DRG are also shown.

| **Gene** | **UTR length** | **miRNA** | **TargetScan prediction** | **mirBase prediction** | **Fold Change SNL L4 ipsi vs contra** | **Fold Change SNL L5 ipsi vs contra** |
| --- | --- | --- | --- | --- | --- | --- |
| Ca -channel, voltage-dependent, alpha 2/delta subunit 1 (*Cacna2d1*) | 281 | miR-301 | no prediction | 19.2273 (highest miRBase score) | -45.3 | -1.8 |
|  |  | miR-148b | no prediction | 18.7696 (second highest miRBase score) | -15.6 | -2.1 |
|  |  | miR-103 | 96th percentile | 17.2496 (high score) | -2.7 | -2.0 |
|  |  | miR-101 | no prediction | 16.5614 | -8.9 | -2.4 |
| Sodium channel, voltage-gated, type XI, alpha subunit (*Nav1.9/ Scn11a*) | 518 | let-7a | 94th percentile | 16.5297 for let-7e (TS groups with let-7a) | -459.7 | -9.7 |
|  |  | miR-125b | no prediction | 17.7028 (high score) | -19.4 | -3.2 |
| Purinergic receptor P2X, ligand-gated ion channel 4 (*P2rx4*) | 471 | miR-133a | 99th percentile | no prediction | -25.9 | -12.0 |
|  |  | miR-20b-5p | 84th percentile | 15.8913 (low score) | -21.3 | -1.1 |
|  |  | miR-20a | (TS groups 20a and 20b-5p) |  | -177.7 | -1.2 |
|  |  | miR-497 | no prediction | 16.0682 | -132.0 | -5.3 |
|  |  | miR-181d | no prediction | 15.9945 (low score) | -3.0 | -1.3 |
