# Supplementary material for: Dynamic Changes in the MicroRNA Expression Profile Reveal Multiple Regulatory Mechanisms in the Spinal Nerve Ligation Model of Neuropathic Pain
Source: PLoS One. 2011 Mar 14;6(3):e17670. doi: 10.1371/journal.pone.0017670 (PMC3056716; doi:10.1371/journal.pone.0017670)
Supplement: Table S2 — A sampling of the lengths of 3′-UTRs used by TargetScan in miRNA target prediction. Two sets of sequences appear: those predicted to have 10 or more (N = 14) 63-set target sites and 14 randomly chosen transcripts predicted to have only one 63-set target site. The rows are sorted by 3′-UTR length and the unit of length is nucleotides. (DOC) [file pone.0017670.s004.doc]

**Table S2.** **A sampling of the lengths of 3’-UTRs used by TargetScan in miRNA target prediction.** Two sets of sequences appear: those predicted to have 10 or more (N=14) 63-set target sites and 14 randomly chosen transcripts predicted to have only one 63-set target site. The rows are sorted by 3’-UTR length and the unit of length is nucleotides.
