# Supplementary material for: Dynamic Changes in the MicroRNA Expression Profile Reveal Multiple Regulatory Mechanisms in the Spinal Nerve Ligation Model of Neuropathic Pain
Source: PLoS One. 2011 Mar 14;6(3):e17670. doi: 10.1371/journal.pone.0017670 (PMC3056716; doi:10.1371/journal.pone.0017670)
Supplement: Table S4 — Ingenuity Pathway Core Analysis. Analysis of functions of 1328 genes with three or more predicted 63-set target sites and a TargetScan context score above the 60th percentile. The listed functions are subcategories under the broader category called “Nervous System Development and Function.” The rows are sorted by p-value and lists with P-values of E-04 and lower are shown. (DOC) [file pone.0017670.s006.doc]

**Table S4.** **Ingenuity Pathway Core Analysis.** Analysis of functions of 1328 genes with three or more predicted 63-set target sites and a TargetScan context score above the 60^th^ percentile. The listed functions are subcategories under the broader category called “Nervous System Development and Function.” The rows are sorted by P-value and lists with P-values of E-04 and lower are shown.
