# Supplementary material for: Dynamic Changes in the MicroRNA Expression Profile Reveal Multiple Regulatory Mechanisms in the Spinal Nerve Ligation Model of Neuropathic Pain
Source: PLoS One. 2011 Mar 14;6(3):e17670. doi: 10.1371/journal.pone.0017670 (PMC3056716; doi:10.1371/journal.pone.0017670)
Supplement: Table S5 — Text mining of miRNAs. 39 microRNAs (of the 63-set) and their literature associated human target genes (Entrez gene symbols) uncovered from a systematic mining of PubMed. Verb-group triplets were identified using regular expressions and a gene thesaurus using the I2E semantic search tool. All entries in bold represent the 10-set. (DOC) [file pone.0017670.s007.doc]

**Table S5.** **Text mining of miRNAs.** 39 microRNAs (of the 63-set) and their literature associated human target genes (Entrez gene symbols) uncovered from a systematic mining of PubMed. Verb-group triplets were identified using regular expressions and a gene thesaurus using the I2E semantic search tool. All entries in bold represent the 10-set.

| **[pt] microRNA** | **# of Publications** | **Entrez Gene Symbol** |
| --- | --- | --- |
| miR-100 | 11 | *FRAP1, KIAA1303* |
| miR-103 | 9 | *FOXJ2* |
| miR-10a | 19 | *HOXA1, HOXA3, HOXD10, HOXD4, USF2* |
| miR-10b | 12 | *HOXD10, RHOC* |
| miR-125b | 37 | *AKT1, EIF4EBP1, ERBB2, ERBB3, IL1RL1, IRF4, LIN28, PRDM1, SMO, ST2, SULT2A1, TNFSF4, TP53, TRIM71* |
| miR-126 | 27 | *CRK, HOXA9, IRS1, PIK3R1, PIK3R2, PLK2, SOX4, SPRED1, VCAM1, VEGFA* |
| miR-127 | 13 | *BCL6, ESRRG, NR0B2* |
| **miR-132** | 14 | *BDNF, CTBP1, GNRH1, MECP2, RICS* |
| miR-133a | 17 | *CCND2, KCNQ1, PKM2, SRF, UCP2* |
| miR-133b | 9 | *KCNQ1, PKM2* |
| **miR-135a** | 5 | *HIF1A* |
| miR-137 | 8 | *CDK6, MITF* |
| miR-142-3p | 14 | *ADCY7, ADCY9* |
| miR-148a | 4 | *NR1I2* |
| miR-181b | 14 | *AICDA* |
| **miR-18a** | 15 | *CTGF, ESR1, RUNX1* |
| miR-19a | 15 | *PTEN, SOCS1* |
| miR-206 | 33 | *ACTB, ESR1, FSTL1, GJA1, IGF1, MSC, MSTN, PTBP1, PTBP2, UTRN* |
| miR-20a | 28 | *APP, CCND1, CDKN1A, E2F1, E2F2, RUNX1* |
| miR-21 | 151 | *BCL2, BMP6, GFI1, IL13, MAPK10, MARCKS, MMP2, NANOG, NFIB, PDCD4, POU5F1, PTEN, RECK, SERPINB5, SOX2, SPRY1, SPRY2, TGFB1, TIMP3, TP53, TPM1* |
| miR-218 | 4 | *LAMB3* |
| **miR-221** | 72 | *BIRC1, CDKN1B, CDKN1C, ESR1, GBA, GBA3, HOXB5, KIT, NOS3* |
| miR-23b | 16 | *GLS, HES1, MDH2, OPRM1, PTGS2, STAR* |
| miR-26a | 18 | *EZH2, SMAD1* |
| miR-27a | 13 | *ABCB1, PHB, RUNX1, ZBTB10* |
| miR-27b | 6 | *CYP1B1* |
| miR-30a-5p | 5 | *BDNF* |
| miR-324-5p | 4 | *GLI1, SMO, VIF* |
| miR-335 | 7 | *SOX4* |
| miR-338 | 7 | *COX4I1, CYCS, PLA2G4B* |
| **miR-34a** | 53 | *BCL2, BIRC5, CCND1, CDK6, E2F3, GRM7, SIRT1, TP53* |
| **miR-34c** | 17 | *PCNA* |
| **miR-378** | 7 | *ENV, FUS, SUFU* |
| miR-659 | 1 | *GRN* |
| miR-9 | 8 | *BACE1, CBX7, CD46, COL9A1, LOC619511, MMP13, NFKB1, NR2E1, NTRK3, PMP22, RCOR1, RUNX1, SYTL4* |
| miR-93 | 15 | *CDKN1B, CDKN1C, E2F1, NUPR1, TP53INP1* |
| miR-Let-7a | 33 | *BCL2, JAK2, REM1, TRIM71* |
| miR-Let-7b | 20 | *BCL7A, CDC25A, CDKN2A, HMGA2, MTPN* |
| miR-Let-7c | 20 | *MYC, TRIM71* |
