# Supplementary material for: Dynamic Changes in the MicroRNA Expression Profile Reveal Multiple Regulatory Mechanisms in the Spinal Nerve Ligation Model of Neuropathic Pain
Source: PLoS One. 2011 Mar 14;6(3):e17670. doi: 10.1371/journal.pone.0017670 (PMC3056716; doi:10.1371/journal.pone.0017670)
Supplement: Table S6 — Partial results from GSEA of 153 miRNA-regulated genes against GO Biological Processes (DAVID). (DOC) [file pone.0017670.s008.doc]

**Table S6.** **Partial results from GSEA of 153 miRNA-regulated genes against GO Biological Processes (DAVID).**

| **GO ID** | **GO Term** | **# genes overlap** | **PValue** |
| --- | --- | --- | --- |
| *DAVID functional group 2* | | | |
| GO:0042127 | regulation of cell proliferation | 32 | 2.6E-17 |
| *DAVID functional group 3* | | | |
| GO:0030154 | cell differentiation | 57 | 7.7E-17 |
| GO:0048468 | cell development | 40 | 9.1E-12 |
| GO:0012501 | programmed cell death | 31 | 4.0E-11 |
| *DAVID functional group 15* | | | |
| GO:0007399 | nervous system development | 23 | 3.5E-06 |
| GO:0022008 | Neurogenesis | 12 | 1.7E-04 |
| GO:0048699 | generation of neurons | 11 | 4.0E-04 |
| GO:0030182 | neuron differentiation | 10 | 5.9E-04 |
| GO:0048666 | neuron development | 6 | 3.5E-02 |
| *DAVID functional group 18* | | | |
| GO:0009605 | response to external stimulus | 20 | 9.2E-06 |
| GO:0006950 | response to stress | 24 | 1.8E-04 |
| GO:0009611 | response to wounding | 12 | 2.4E-03 |
| GO:0050896 | response to stimulus | 43 | 5.1E-03 |
| GO:0006954 | inflammatory response | 9 | 7.5E-03 |
| GO:0006952 | defense response | 11 | 4.5E-02 |
| *DAVID functional group 74* | | | |
| GO:0048666 | neuron development | 6 | 3.5E-02 |
| GO:0031175 | neurite development | 3 | 4.6E-01 |
| GO:0030030 | cell projection organization and biogenesis | 3 | 6.5E-01 |
| GO:0048858 | cell projection morphogenesis | 3 | 6.5E-01 |
| GO:0032990 | cell part morphogenesis | 3 | 6.5E-01 |
