# Supplementary material for: Dynamic Changes in the MicroRNA Expression Profile Reveal Multiple Regulatory Mechanisms in the Spinal Nerve Ligation Model of Neuropathic Pain
Source: PLoS One. 2011 Mar 14;6(3):e17670. doi: 10.1371/journal.pone.0017670 (PMC3056716; doi:10.1371/journal.pone.0017670)
Supplement: Table S7 — Neurite or axonal phenotypes regulated by the 63-set associated gene. (DOC) [file pone.0017670.s009.doc]

**Table S7.** **Neurite or axonal phenotypes regulated by the 63-set associated gene.**

| **Neurite/Axonal Condition** | **Entrezgene Symbol** | **[pt] microRNA** |
| --- | --- | --- |
| Axonal growth | *AKT1* | miR-125b |
|  |  | miR-126 |
|  | *CMM* | miR-133b |
|  | *ERBB2* | miR-125b |
|  | *LOC619511* | miR-9 |
|  | *STAT3* | miR-21 |
|  | *TNC* | miR-335 |
| Axonal outgrowth | *CRK* | miR-126 |
|  | *ESR2* | miR-206 |
|  | *NFIB* | miR-21 |
|  | *PTEN* | miR-21 |
|  | *TNC* | miR-335 |
|  | *TNF* | miR-125b |
| Axonal regeneration | *AGT* | miR-21 |
|  | *AKT1* | miR-125b |
|  |  | miR-126 |
|  | *FHIT* | miR-21 |
|  | *PTEN* | miR-21 |
|  | *TCHP* | miR-21 |
|  | *TNC* | miR-335 |
|  | *TNF* | miR-125b |
|  | *TP53* | miR-21 |
|  |  | **miR-34a** |
|  |  | **miR-34c** |
| Axonal sprouting | *ERBB2* | miR-125b |
|  | *TAC1* | miR-206 |
| Neurite growth | *AKT1* | miR-125b |
|  |  | miR-126 |
|  | *ESR2* | miR-206 |
|  | *MYC* | miR-let-7C |
|  | *PSEN1* | miR-9 |
|  | *RICS* | **miR-132** |
| Neurite outgrowth | *AKT1* | miR-125b |
|  |  | miR-126 |
|  | *CCND1* | **miR-34a** |
|  | *CRK* | miR-126 |
|  | *E2F1* | miR-20a |
|  | *ERBB2* | miR-125b |
|  | *ESR1* | miR-206 |
|  | *FRAP1* | miR-100 |
|  | *HES1* | miR-23b |
|  | *KIT* | miR-221 |
|  | *NFIB* | miR-21 |
|  | *PSEN1* | miR-9 |
|  | *PTEN* | miR-21 |
|  | *RICS* | **miR-132** |
|  | *SPRY2* | miR-21 |
|  | *STAT3* | miR-21 |
|  | *SRF* | miR-133b |
|  | *TAC1* | miR-206 |
|  | *TCHP* | miR-21 |
|  | *TNC* | miR-335 |
|  | *TP53* | miR-21 |
|  |  | **miR-34a** |
|  |  | **miR-34c** |
|  | *VCAM1* | miR-126 |
|  |  | miR-126 |
