# Supplementary material for: Dynamic Changes in the MicroRNA Expression Profile Reveal Multiple Regulatory Mechanisms in the Spinal Nerve Ligation Model of Neuropathic Pain
Source: PLoS One. 2011 Mar 14;6(3):e17670. doi: 10.1371/journal.pone.0017670 (PMC3056716; doi:10.1371/journal.pone.0017670)
Supplement: Table S9 — microRNA nomenclature. (DOC) [file pone.0017670.s011.doc]

**Table S9 microRNA nomenclature**

| ABI Name | TargetScan Name |
| --- | --- |
| miR-10a | miR-10a-5p |
| miR-125b | miR-125b-5p |
| miR-148b | miR-148-3p |
| miR-339 | miR-339-5p |
| miR-301 | miR-301a |
| miR-92 | miR-92a |
| miR-30a-5p | miR-30a |
| miR-299-5p | miR-299 |
| miR-101 | miR-101a |
| miR-7 | miR-7a |
| let-7g | Not found |

Table 1:miRNA name conversion between the ABI platform and TargetScan

| ABI Paralogs | TargetScan Representative used in this study |
| --- | --- |
| miR-30a, miR-30d | miR-30a |
| miR-34a, miR-34c | miR-34a |
| miR-133a, miR-133b | miR-133a |
| miR-148a, miR-148b-3p | miR-148b-3p |
| miR-26a, miR-26b | miR-26a |
| let-7a, let-7b, let-7c, let-7d, let-7e | let-7a |
| miR-181b, miR-181c, miR-181d | miR-181b |
| miR-19a, miR-19b | miR-19a |
| miR-27a, miR-27b | miR-27a |
| miR-20a, miR-20b, miR-93 | miR-20a |

Table 2: miRNA Paralog representative used by TargetScan

After name changes and the elimination of paralogous members from the ABI list of 63 miRNAs, the following list of 43 miRNA names was used in mining the TargetScan database:

let-7a

miR-100

miR-103

miR-10a-5p

miR-10b

miR-125b-5p

miR-126

miR-127

miR-132

miR-133a

miR-135a

miR-137

miR-142-3p

miR-142-5p

miR-148b-3p

miR-17-3p

miR-181b

miR-18a

miR-190

miR-19a

miR-206

miR-20a

miR-21

miR-218

miR-221

miR-23b

miR-26a

miR-27a

miR-299

miR-301a

miR-30a

miR-324-5p

miR-335

miR-338

miR-339-5p

miR-34a

miR-369-5p

miR-378

miR-383

miR-409-5p

miR-497

miR-9

miR-92a
